# Integrative multiomic characterization of the methylome and restriction-modification repertoire of *Mycoplasma hyorhinis* SK76

**DOI:** 10.3389/fmicb.2026.1950185

**Published:** 2026-10-22

**Authors:** Elisabet Frutos-Grilo, Matilde D’Angelo, Miquel Anglada-Girotto, Luis Serrano

**Affiliations:** 1 Centre for Genomic Regulation (CRG), The Barcelona Institute of Science and Technology, Barcelona, Spain; 2 Universitat Pompeu Fabra (UPF), Barcelona, Spain; 3 ICREA, Barcelona, Spain

**Keywords:** methylation, methyltransferase, *Mycoplasma hyorhinis*, nanopore sequencing, restriction-modification system

## Abstract

Restriction-modification (R-M) systems are widespread in *Mycoplasma* species, contributing to defense against foreign DNA and epigenetic regulation. However, the limited genetic tractability of Mollicutes hinders the functional characterization of R-M systems. Here, we characterized the R-M repertoire of *Mycoplasma hyorhinis* SK76 by integrating comparative genomics, structural modeling, quantitative proteomics, and genome-wide methylome profiling. We found that SK76 encodes Type I, Type II and Type III R-M systems with some duplicated components. For Type I, HsdS2 was predicted to recognize a bipartite TA-N₆-ATC motif, which was detected as a secondary 6mA-associated methylation signature, whereas the remaining HsdS variants displayed features consistent with reduced functionality. Among the Type II R-M system, Mhy3 and its cognate endonuclease End3 were associated with the 5mC motif GATC, while the remaining predicted methyltransferases lacked both protein expression and corresponding methylation signatures. Regarding Type III, Mod1 was identified as the principal active adenine methyltransferase responsible for the asymmetric GGAT-centered methylation pattern, whereas additional Mod homologues likely represent phase-variable or conditionally regulated elements with limited activity. Overall, the functional methylome of *M. hyorhinis* represents only a subset of its genomic R-M repertoire, underscoring the value of integrating genomic, proteomic and methylome data to define active epigenetic systems.

## Introduction

*Mycoplasma hyorhinis* is a wall-less Mollicute bacterium primarily recognized as a porcine pathogen and a frequent contaminant of mammalian cell cultures (Yang et al., 2025; Liu et al., 2025). Beyond its veterinary relevance, *M. hyorhinis* has gained increasing biomedical attention due to its recurrent detection in human tumors, including gastric (56%), colorectal (55%), and lung (53%) cancers (Huang et al., 2001). Persistent infection and different bacterial proteins (Gomersall et al., 2015) have been implicated in inflammatory signaling, enhanced cell migration, and tumor progression (Kim et al., 2023). Importantly, experimental evidence indicates that *M. hyorhinis* restriction-modification (R-M) systems and helicases contribute to bacterial virulence and host interactions in pigs (Obregon-Gutierrez et al., 2025). Additionally, Type II DNA methyltransferases from *M. hyorhinis* have been shown to modify cytosine residues within the host genome following invasion, altering methylation patterns in genes associated with proliferation and carcinogenesis in humans (Chernov et al., 2015). These findings suggest that DNA methylation plays an important role not only in bacterial genome defense but potentially also in bacteria-host interaction and pathogenicity.

Despite this relevant evidence, the full repertoire of DNA methyltransferases encoded by *M. hyorhinis* remains incompletely defined. In bacteria, DNA methylation is predominantly mediated by R-M systems, which constitutes one of the most widespread mechanisms for distinguishing self from non-self DNA. These systems typically comprise a restriction endonuclease (R) that cleaves unmethylated foreign DNA and a cognate DNA methyltransferase (M) that modifies specific sequence motifs within the host genome, thereby protecting self-DNA from cleavage. Beyond their canonical role in defense against exogenous DNA, R-M systems have also been implicated in the regulation of gene expression, genome stability, horizontal gene transfer, and bacterial adaptation (Vasu and Nagaraja, 2013; Oliveira et al., 2014). R-M-associated methyltransferases modify specific cytosine or adenine residues, generating epigenetic marks such as 5-methylcytosine (5mC), 4-methylcytosine (4mC), and N6-methyladenine (6 mA) (Oliveira and Fang, 2021). R-M systems with methylation activity are classified into three major types (I-III) based on differences in subunit composition, DNA recognition, and catalytic requirements (Bower et al., 2018).

Type I systems are multi-subunit complexes composed of a methyltransferase (HsdM), a specificity subunit (HsdS), and a restriction endonuclease (HsdR). DNA sequence recognition is determined by HsdS, whereas HsdM methylates DNA within an HsdM₂-HsdS complex. The HsdR subunit, when assembled with HsdM and HsdS into the R₂M₂S restriction-competent complex, cleaves unmethylated target DNA (Murray, 2000). Type II is the simplest and most abundant among the various R-M systems. This type typically functions by using two entirely independent enzymes: the restriction endonuclease, which cuts double-stranded DNA (dsDNA) non-methylated targeted sequences, and the methyltransferase, which modifies DNA target sequences to protect them from cleavage. Type III systems consist of a methyltransferase subunit (Mod) and a restriction endonuclease (Res). Mod is sufficient for DNA methylation, commonly functioning as a homodimer (M₂), whereas DNA cleavage of non-methylated DNA requires association with the Res subunit. In several bacterial species, Type III methyltransferases have been linked not only to host defense but also to phase-variable gene regulation and epigenetic control (Kang et al., 2025). In addition to these three classical methylation-based R-M systems, Type IV R-M has also been described. Type IV system recognizes and cleaves modified DNA and generally lack a cognate protective methyltransferase (Vasu and Nagaraja, 2013). Because the present study focuses on DNA methylation, the methyltransferases responsible for these modifications, and, where applicable, their cognate restriction enzymes, we concentrate on Type I, II, and III R-M systems.

Despite evidence that *M. hyorhinis* encodes several putative DNA methyltransferases, the diversity and sequence specificity of its DNA methylation systems remain incompletely characterized. The type II DNA methylation systems have been investigated by heterologous expression of the three putative *M. hyorhinis* SK76 methyltransferases (Mhy) enzymes in an *Escherichia coli* strain lacking the endogenous cytosine-specific Dcm methyltransferase. This was followed by bisulfite-based analyses identifying 5mC methylation at CATG and CG DNA motifs (Gonzalez et al., 2015) (methylated base is underlined in motif sequences throughout the manuscript). However, bisulfite sequencing efficiently detects 5mC, but it cannot reliably distinguish 4mC, which may be misidentified as 5mC, and it does not detect 6 mA. Consequently, bacterial methylomes characterized solely by bisulfite sequencing may underestimate the diversity of DNA methylation marks and biasing interpretations of R-M system functionality and epigenetic regulation. Recent advances in third-generation sequencing technologies, including single-molecule real-time sequencing and Oxford Nanopore sequencing, now enable the direct detection of multiple DNA base modifications at single-molecule resolution (i.e., 6 mA, 5mC, and 4mC) without chemical conversion or amplification bias (Sigurpalsdottir et al., 2024; Gouil and Keniry, 2019). Also, it has been shown that *M. hyorhinis* ATCC 17981 strain has a 6 mA modification in, but no motif or responsible system was identified (Razin and Razin, 1980). This underscores the need for a comprehensive, motif-resolved characterization of the *M. hyorhinis* methylome using technologies capable of detecting multiple classes of DNA modifications.

In this study, we provide a comprehensive characterization of the DNA methylation landscape of *M. hyorhinis* SK76. By integrating genomic annotation, structural modeling, proteomics, and Oxford Nanopore sequencing, we defined the repertoire and organization of its restriction-modification systems, identified the components expressed under the conditions analysed, and linked these systems to the methylation patterns detected across the genome. This integrative analysis also enabled the refinement of existing annotations and provided a unified view of the three classes of restriction-modification systems present in this strain. Together, our findings establish a framework for understanding DNA modification in *M. hyorhinis* and provide a basis for future studies on its epigenetic regulation, host interactions, and pathogenicity.

## Materials and methods

### Strains

*M. hyorhinis* strain SK76 was kindly provided by Kim S. Wise (University of Missouri, Columbia, MO) (Goodison et al., 2013). Planktonic Mycoplasma was grown in the SP4 broth (Mizutani et al., 2025) at 37 °C with shaking at 180 rpm, harvested after 48 h for exponential-phase cultures or after 96 h for stationary-phase cultures. *E. coli* DH5α and *dam*^−^/*dcm*^−^ strains were obtained commercially (ThermoFisher and NEB, respectively) and cultured in LB medium supplemented with 34 μg/mL chloramphenicol to maintain transposon stability in the mutant strain.

### Identification of restriction-modification system homologs

Putative R-M system components were identified in the *M. hyorhinis* SK76 genome (taxID 1,118,964) through a domain-based search using the Pfam Database. Hidden Markov Model profiles corresponding to known R-M enzyme families were retrieved from Pfam and queried against the predicted proteome of NC_019552.1 using hmmscan (HMMER v3.4) (Potter et al., 2018) using default parameters. All significant hits (e-value < 1e-10) were compiled, and candidates were manually curated to exclude truncated sequences or annotations consistent with pseudogenes, ensuring that only intact open reading frames were retained. The following Pfam domains were used to identify specific subunits: Type I methyltransferases (PF02384), Type I specificity subunits (PF01420), Type II methyltransferases (PF00145), Type III methyltransferases (Mod subunits) (PF01555), and Type III restriction endonuclease (PF04851). For Type II restriction enzymes, there is no specific PFAM capable of identifying them; however, End1-3 have been identified by proximity of these genes to their associated Type II methyltransferase genes.

To further validate all candidates, protein sequences were aligned using Clustal Omega (version 1.2.4) (Madeira et al., 2024) with default parameters using a reviewed Dataset of proteins containing the corresponding PFAM from InterPro. Alignments were inspected to verify the presence of conserved catalytic motifs and to assess whether protein length and domain organization were consistent with functional homologs. Only sequences meeting these criteria were retained to identify the R-M subunits for subsequent analyses. Jalview was used to visualize the alignments, coloring sequences according to identity percentage, and WebLogo3 (Crooks et al., 2004) was used to generate sequence logos representing conserved motifs across the alignments. Simple sequence repeats (SSRs) were identified in methyltransferase gene sequences using MISA (Microsatellite Identification Tool) (Beier et al., 2017) from FASTA-formatted inputs. Search parameters were set to detect mononucleotide repeats ≥5, dinucleotide repeats ≥3, and tri- to hexanucleotide repeats ≥3, allowing compound SSRs with interruptions up to 100 bp. SSRs were classified by motif type and frequency based on the MISA output files (Supplementary Data Sheet S1).

### Analysis of 5′ upstream regions for promoter search

Transcription start sites and translation initiation sites were predicted by analyzing the 80 bp 5′ upstream regions, following previously described (Fisunov et al., 2016). Putative −10 (Pribnow) promoter elements were identified within these regions. Because canonical −35 elements can be poorly conserved or absent in several Mycoplasma species (Hyman et al., 1988; Weber et al., 2012; Pich et al., 2006), their presence was not required for promoter assignment. In addition, repeat regions within these sequences were examined to identify potential short hairpin structures using Palindromic Sequence Finder server^1^.

### Structural modeling using AlphaFold 3

Structural predictions of the candidate restriction-modification proteins were generated using AlphaFold Protein Structure Prediction and AlphaFold-Multimer (Jumper et al., 2021; Evans et al., 2021) under its non-commercial license. Protein-DNA complexes were modeled with AlphaFold 3 using the corresponding protein sequence(s) and DNA substrates containing the methylation motifs identified in the methylome analysis. Model confidence was evaluated using pLDDT, pTM and ipTM scores, which are listed in Supplementary Data Sheet S2. For each prediction, all default models generated by the AlphaFold 3 server were examined, and the highest-ranking model was selected for subsequent structural analyses. Models were inspected in PyMOL and compared with experimentally determined DNA methyltransferase structures to assess domain architecture and catalytic motifs arrangements, using representative structures for Type I, Type II and Type III methyltransferases as indicated in each figure. Structural superpositions were performed in PyMOL using the align command, which combines sequence alignment, structural superposition and iterative outlier rejection. The resulting root mean square deviation (RMSD) values were used to evaluate structural similarity between predicted and reference models.

For Table 1, an experimentally characterized EcoKI Type I complex (UniProt IDs P08956 for HsdR and P08957 for HsdM) was included as a positive control to provide a reference for a validated Type I complex, whereas the non-cognate 2Mhy1-HsdS1 pair was used as a negative control. This negative control retains the same species background while combining proteins that are not expected to form a physiological Type I M₂S complex, allowing structural compatibility to be distinguished from species-specific effects.

**Table 1:** Predicted M_2_S assemblies for each HsdS variant with corresponding predicted (pTM) and interface (ipTM) template modeling scores.

HsdS variant	ipTM	pTM
HsdS1	0.65	0.65
HsdS2	0.69	0.69
HsdS3	0.68	0.69
Negative control (Mhy1)	0.21	0.43
Positive control (EcoKI)	0.42	0.47

Negative and positive controls are included (Mhy1 with HsdS1, and EcoKI complete system, respectively).

### Genomic DNA extraction and Oxford Nanopore sequencing

Genomic DNA was extracted from *M. hyorhinis* SK76 cell pellets obtained from 30 mL stationary-phase cultures using the Tissue & Cell Lysis Solution and MPC Protein Precipitation Reagent (Lucigen), according to the manufacturer’s instructions. DNA concentration and purity were assessed using a NanoDrop spectrophotometer, and DNA integrity was verified by 1% agarose gel electrophoresis. DNA samples were stored at −20 °C until further use.

Library preparation and sequencing were performed at the CRG Genomics Unit. Genomic DNA from three biological replicates of *M. hyorhinis* SK76 was pooled before library preparation and processed using the Rapid Barcoding Kit 24 V14 (SQK-RBK114.24). The pooled SK76 sample was assigned to barcode RB01, combined with other barcoded samples and sequenced on an Oxford Nanopore Technologies GridION Mk1 platform using an R10.4.1 flow cell (FLO-MIN114) at a translocation speed of 400 bases s⁻^1^. Sequencing was performed for approximately 53 h. Basecalling and demultiplexing were performed using the onboard MinKNOW software with the super-accurate basecalling model. Reads shorter than 200 bp or with a quality score below 10 were excluded. Barcode trimming and mid-read barcode filtering were enabled, using minimum barcode and mid-read barcode scores of 80%. Following demultiplexing, 308,800 reads assigned to barcode RB01 passed the quality filters, whereas 15,700 reads failed. The non-failed reads assigned to RB01 were used for downstream methylome analysis. The raw Oxford Nanopore sequencing data generated in this study have been deposited in the European Nucleotide Archive (ENA) under Project accession number PRJEB122860.

### Methylation detection and motif analysis

DNA methylation in *M. hyorhinis* SK76 was analyzed using Nanopore sequencing Data. Mapped reads (BAM format) were processed with MicrobeMod (Galeone et al., 2025) to detect methylated bases and identify methylation motifs. The analysis was run with the following parameters: minimum strand coverage of 3, methylation confidence threshold of 0.66, percent methylation cutoff of 0.0, and motif discovery via STREME with thresholds of 0, 0.5, 0.7, and 0.9 for comparative purposes. Supplementary Data Sheet S3 compiles processed results.

### LC–MS/MS analysis and database searching

*M. hyorhinis* SK76 cultures were grown as previously described, harvested at exponential and stationary phases, and washed with PBS. Two biological replicates were prepared, and protein concentrations were determined using the Pierce BCA Protein kit following the manufacturer’s protocol. Samples were digested in solution using sequential LysC and trypsin treatment, reduced with dithiothreitol, and alkylated with iodoacetamide. Peptides were desalted prior to LC–MS/MS analysis.

Peptide separation was performed on a 50 cm μPAC™ Neo column using a Vanquish Neo liquid chromatographer, coupled to an Orbitrap Astral mass spectrometer. Data-independent acquisition (DIA) was used, with full MS scans over m/z 380–980 and 300 consecutive 2 Th isolation windows for fragmentation. Peptide ions were fragmented using HCD with normalized collision energy of 25%, and MS2 detected in the Astral with a maximum injection time of 3 ms. Bovine serum albumin digests were analyzed between samples to monitor instrument performance. Raw spectra were analyzed with DIA-NN (v2.2) using a predicted spectral library for *M. hyorhinis* plus common contaminants. Trypsin digestion, variable oxidation (Met) and N-terminal acetylation, and fixed carbamidomethylation (Cys) were applied. FDR was set to 1% at precursor and protein levels, and match-between-runs and high-precision quantification strategies were employed. Protein inference used heuristic assignment from FASTA names. Proteins for which no shared or specific proteotypic peptides were detected were classified as “not detected” under the conditions analyzed. This classification does not exclude low-level expression below the sensitivity of the MS workflow. The mass spectrometry proteomics data have been deposited to the ProteomeXchange Consortium via the PRIDE (Perez-Riverol et al., 2025) partner repository with the dataset identifier PXD080153.

All Data and analysis are compiled in Supplementary Data Sheet S4. For full experimental details, including buffer compositions, gradient settings, acquisition parameters, and all DIA-NN settings, see Supplementary Data Sheet S5.

### Phylogenetic tree of type III methylases

Protein sequences annotated as Type III DNA methyltransferases were retrieved from UniProtKB (September 2025) with a minimum length of 400 amino acids to exclude truncated or fragmentary entries. Redundant sequences sharing ≥90% identity and entries lacking clear species annotation were removed. The three *M. hyorhinis* SK76 Mod methyltransferases were added, resulting in a final curated Dataset of 36 sequences. Multiple sequence alignment was performed using T-Coffee, and maximum-likelihood phylogenetic trees were constructed with IQ-TREE v2.1.2 (Minh et al., 2020) with 100 bootstrap replicates. Trees were visualized using iTOL v7^2^.

To contextualize the Mod phylogeny relative to species relationships, RecA sequences from each organism were aligned and analyzed under the same pipeline to generate a reference species tree, allowing evaluation of congruence between gene and species phylogenies.

### Conservation of genes across *M. hyorhinis* strains

A total of 23 *M. hyorhinis* strains with complete or high-quality genome sequences were selected from NCBI Database to allow reliable comparison of R-M system genes across the species. This includes SK76, JF5820 derivatives, and diverse clinical and laboratory isolates. For each strain, coding sequences (CDS) were retrieved from NCBI. Local BLAST searches were performed using default parameters to identify homologs of SK76 R-M system genes. BLAST results are provided in Supplementary Data Sheet S6. For the comparative figures, a gene was considered present if sequence identity exceeded 90% with coverage >75%, moderately conserved if identity was 70–90%, and absent if identity was <70% or coverage <75%.

### Protein sequence alignment and conserved motif analysis

Conserved motifs in R-M proteins were analyzed using curated reference datasets. Reviewed Pfam-associated sequences were retrieved for HsdR (PF04313), HsdM (PF02384), Mhy methyltransferases (PF00145), and Res (PF04851). For End3, sequences containing the MutH-associated domain PF02976 were selected to obtain a more specific Sau3IR-related dataset (Friedhoff et al., 2001); broader PD-(D/E)XK-associated Pfam domains were not used because they include many unrelated proteins. For Type III Mod proteins, reviewed UniProtKB entries annotated as Type III DNA methyltransferases were used instead of a Pfam-based dataset. *M. hyorhinis* SK76 sequences were added to the corresponding datasets and aligned with Clustal Omega (v1.2.4) (Madeira et al., 2024) with default parameters. Conserved catalytic, cofactor-binding, and ATPase regions were inspected, and sequence logos were generated with WebLogo3. Complete datasets, alignments, and logos are provided in Supplementary Data Sheet S7.

## Results

### *In silico* characterization and proteomic evidence for type I R-M components in *Mycoplasma hyorhinis* SK76

A complete Type I R-M system comprising the canonical HsdR-HsdM-HsdS architecture was identified in the *M. hyorhinis* SK76 genome, including one HsdM methyltransferase, one HsdR restriction subunit, and three putative HsdS specificity subunits (Figure 1). The *hsdS1* and *hsdS2* genes are arranged in a compact, oppositely oriented configuration with a 25 bp overlap, whereas the *hsdS3* variant is located at a distant chromosomal locus. By sequence analysis of the *hsdS1* gene, we found that the annotations in SK76 NC_019552.1 (265023–266,237) and CP003914.1 (265212–266,237) incorrectly defined the start codon. The actual start codon is a canonical methionine located upstream of both variants, with a putative promoter region included (Supplementary Data Sheet S8), placing the predicted *hsdS1* locus at positions 265,167–266,237. All three HsdS proteins contain two N-6 DNA methylase specificity domains (Supplementary Data Sheet S9). AlphaFold 3 structural modeling (Figure 2A) showed similar overall folds across the three variants, comprising two globular DNA target recognition domains (TRD1 and TRD2) connected by an α-helical linker. Structural superposition showed similar spatial positioning of both TRDs, with RMSDs of 3.2 A for HsdS2 and 2.4 A for HsdS3 relative to HsdS1. Sequence identity analysis revealed substantial differences among the three proteins (Figure 2B; Supplementary Data Sheet S10).

**Figure 1:**
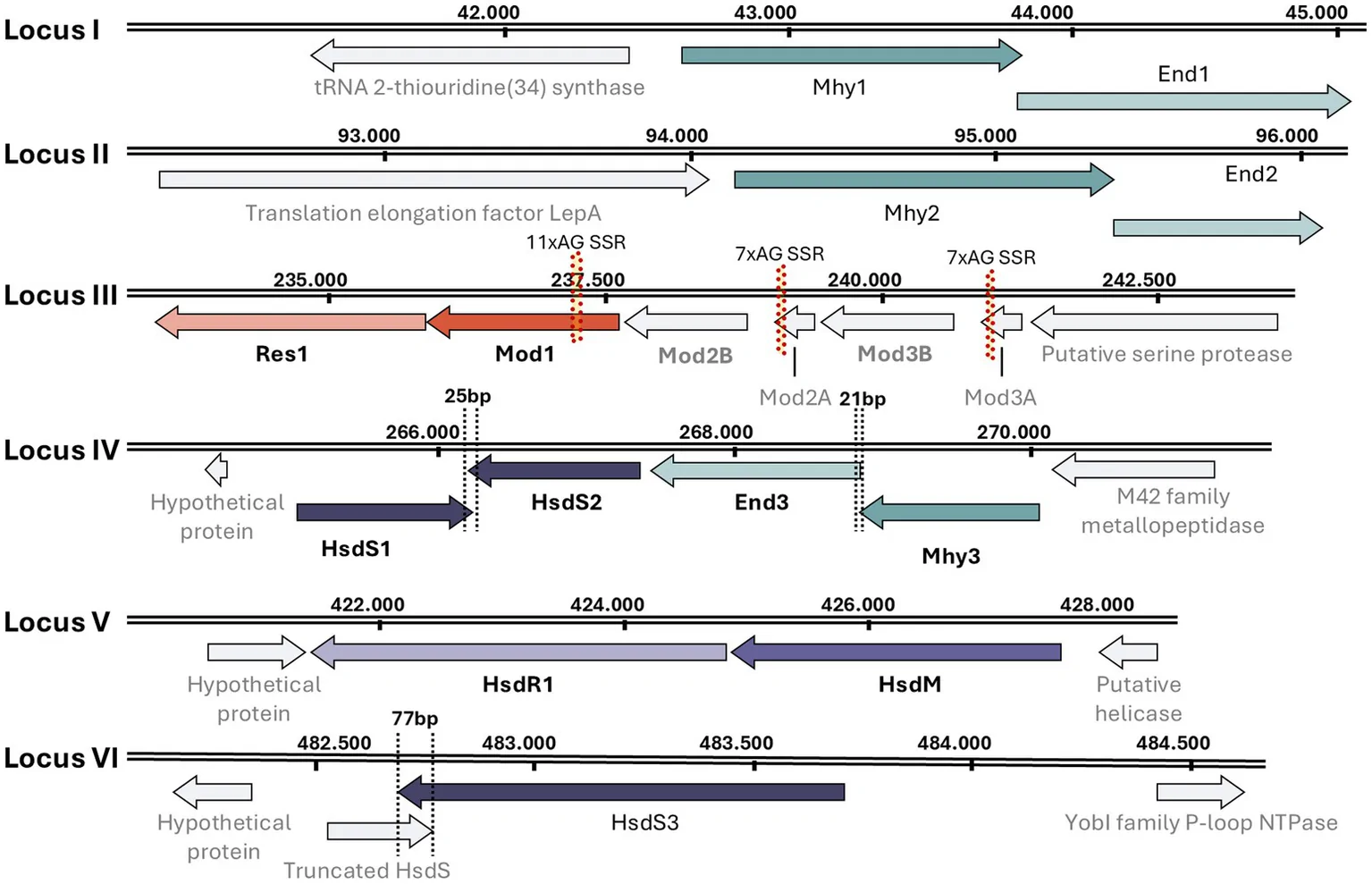
R-M system organization in *M. hyorhinis* SK76. Genomic organization of predicted R-M systems. Arrows indicate coding sequences and transcription direction. Type I genes are purple, Type II green, and Type III red tones. Bold names indicate proteins detected in the proteome (Table 2). SSRs are shown as red boxes with yellow shading. Neighboring genes are shown in gray. Annotations were manually curated based on RefSeq (NC_019552.1) and GenBank (CP003914.1).

**Figure 2:**
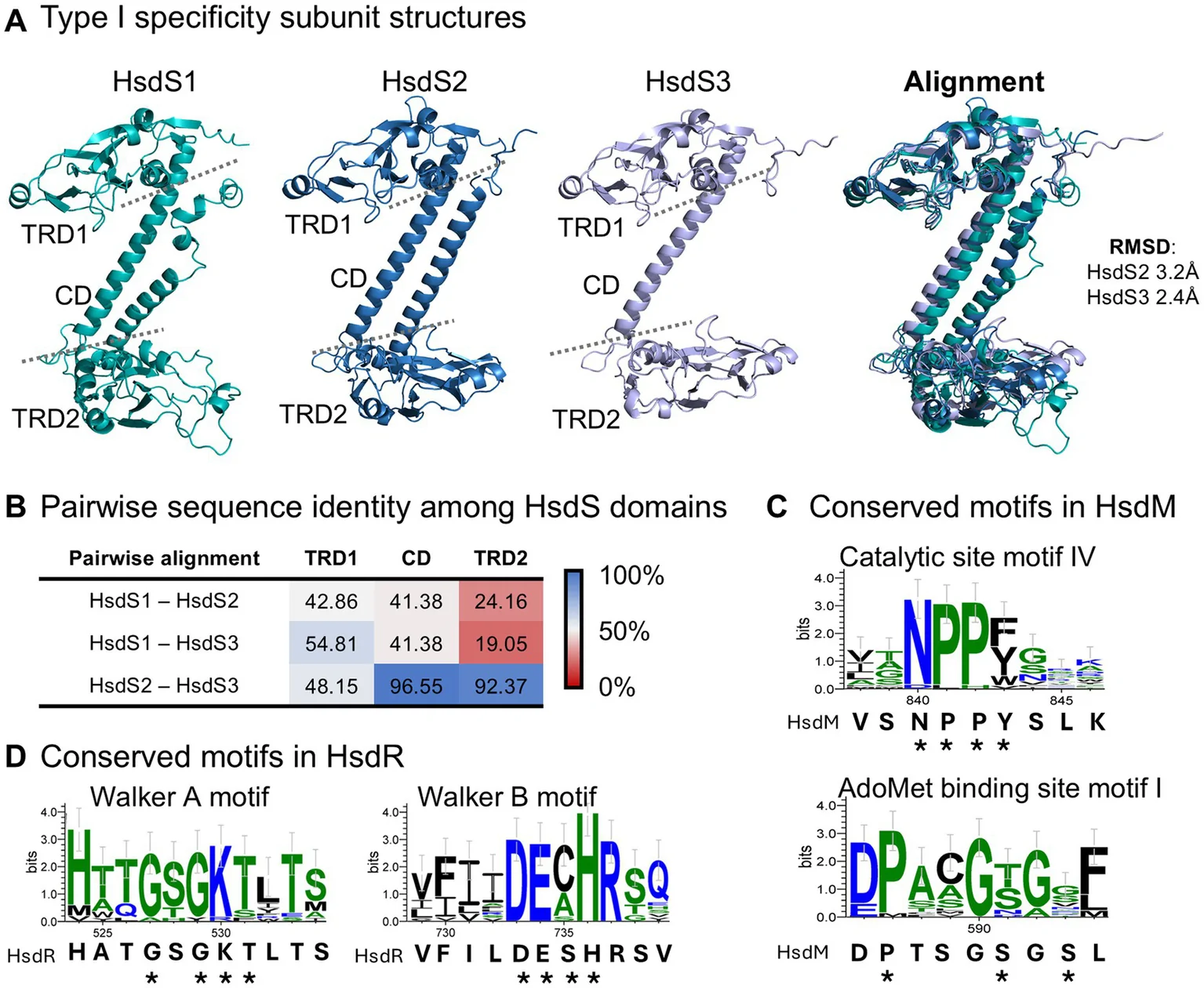
Structural and sequence analysis of Type I R-M system subunits. **(A)** Predicted 3D structures of HsdS1, HsdS2, and HsdS3 subunits showing the N-terminal (TRD1), central (CD), and C-terminal (TRD2) domains. The alignment on the right illustrates structural similarity and differences among the three variants. Root-mean-square deviation (RMSD) values against HsdS1 are indicated. **(B)** Pairwise sequence identity (%) among the HsdS domains (TRD1, CD, TRD2). **(C,D)** Sequence logos highlighting conserved motifs in *M. hyorhinis* HsdM and HsdR. HsdM logos show catalytic motif IV and AdoMet-binding motif I, whereas HsdR logos show the Walker A and B motifs. Asterisks indicate residues involved in catalysis, cofactor binding, or ATP hydrolysis.

HsdS2 and HsdS3 shared 96.55% identity across the central conserved region and 92.37% across TRD2, but only 48.15% within TRD1. HsdS1 was more divergent across all analyzed regions (TRD1: 42.86–54.81%; central domain: 41.38%; TRD2: 19.05–24.16%) (Figure 2B). In this respect, HsdS2 contained the complete coiled-coil central helical region, whereas HsdS1 contained a shorter helix and HsdS3 lacked the second alpha-helix of this region (Figure 2A), suggesting that it could be a pseudogene.

Sequence analysis of HsdM identified the conserved NPPY catalytic motif and a Rossmann-like AdoMet-binding core (Figure 2C; Supplementary Data Sheet S9) supporting the catalytic competence of the Type I methyltransferase. HsdR contained the PF04313 Type I restriction enzyme R domain together with DEAD/DEAH-box helicase motifs (Figure 2D; Supplementary Data Sheet S9) consistent with ATP-dependent DNA translocation and restriction activity characteristic of canonical Type I systems (van Noort, 2004).

Proteomic analysis detected HsdS1 and HsdS2 in both exponential and stationary phases, whereas no unique peptides could be confidently assigned to HsdS3 (Table 2). HsdS1 showed a mean protein intensity approximately 3 log₂ units lower than HsdS2. Promoter analysis identified a Pribnow box (PB) and Shine-Dalgarno (SD) motif upstream of all three *hsdS* genes (Supplementary Data Sheet S8). HsdM and HsdR were also detected by proteomics (Table 2) in agreement with genomic predictions based on the presence of both PB and SD motifs upstream of their coding sequences (Supplementary Data Sheet S8). HsdS2, HsdM and HsdR showed lower abundance in stationary than in exponential phase.

**Table 2:** Proteomic detection of R-M system components in *M. hyorhinis* SK76.

RefSeq/GeneBank	Protein	Mean intensity Exp	Mean intensity Stat	Detection	Found peptides
WP_052306315/AFX74154	Type I HsdS specificity subunit (HsdS1)	20.7	20.4	1/2 Exp, 1/2 Stat	8
WP_015084083/AFX74155	Type I HsdS specificity subunit (HsdS2)	24.3	23.1	1/2 Exp, 1/2 Stat	14
WP_015084170/AFX74343	Type I HsdS specificity subunit (Hsd3)	NA	NA	Unconfirmed	0
WP_015084138/AFX74293	Type I HsdM methyltransferase (HsdM1)	25.2 ± 1.2	26.1 ± 0.7	2/2 Exp, 2/2 Stat	57
WP_014335478/AFX74292	Type I HsdR restriction subunit (HsdR1)	27.7 ± 0.7	28.7 ± 0.6	2/2 Exp, 2/2 Stat	118
WP_013301872/AFX73971	Type II cytosine methyltransferase (Mhy1)	ND	ND	Not detected	0
WP_013301873/AFX73972	Type II restriction (End1)	ND	ND	Not detected	0
WP_015084000/AFX74013	Type II cytosine methyltransferase (Mhy2)	ND	ND	Not detected	0
WP_014335361/AFX74014	Type II restriction (End2)	13.2	13.9	1/2 Exp, 1/2 Stat	2
WP_015084085/AFX74157	Type II cytosine methyltransferase (Mhy3)	27.2 ± 0.9	27.3 ± 1.1	2/2 Exp, 2/2 Stat	27
WP_015084084/AFX74156	Type II restriction (End3)	22.4 ± 0.7	23.5 ± 0.8	2/2 Exp, 2/2 Stat	31
WP_015084066/AFX74131	Type III Mod methyltransferase (Mod1)	23.4 ± 1.3	25.1 ± 0.6	2/2 Exp, 2/2 Stat	23
WP_420704347/AFX74132	Type III Mod methyltransferase (Mod2B)	22.4 ± 3.3	26.2	2/2 Exp, 1/2 Stat	19
NA/AFX74133	Type III Mod methyltransferase (Mod2A)	NA	NA	Unconfirmed	0
WP_337972343/AFX74134	Type III Mod methyltransferase (Mod3B)	20.4	22.1 ± 0.4	1/2 Exp, 2/2 Stat	9
NA/AFX74135	Type III Mod methyltransferase (Mod3A)	NA	NA	Unconfirmed	0
WP_015084065/AFX74130	Type III Res restriction subunit (Res1)	23.6 ± 1.4	25.5 ± 0.8	2/2 Exp, 2/2 Stat	52

Normalized log2 protein intensities are shown for two biological replicates in exponential (Exp) and stationary (Stat) growth phases. Mean ± SD values were calculated when the protein was detected in both biological replicates. When a protein was detected in only one replicate, the normalized intensity of the detected replicate is reported without SD. *Detection* indicates the number of replicates in which the protein was identified. *Unconfirmed* indicates that the detected peptides were not unique to the specific protein, whereas *Not detected* indicates that no shared or specific proteotypic peptides were detected. NA: Not Applicable.

To assess the structural compatibility of HsdM with the different HsdS variants, AlphaFold-Multimer models of M₂S assemblies were generated (Table 1). Model confidence was evaluated using pTM and ipTM, which reflect confidence in the overall complex architecture and inter-chain interfaces, respectively. All three M₂S combinations showed similar pTM/ipTM scores (Table 1), higher than the positive control (EcoKI complex) and much higher than the negative one (non-cognate 2Mhy1-HsdS1). Although alternative assemblies involving HsdS1, HsdS2, or HsdS3 cannot be excluded, the low abundance of HsdS1 and the truncation of HsdS3 support HsdS2 as the most likely primary specificity subunit of the Type I system.

### *In silico* characterization and proteomic evidence for type II R-M components in *Mycoplasma hyorhinis* SK76

Three putative Type II cytosine methyltransferases (Mhy1, Mhy2 and Mhy3) were identified at independent chromosomal loci (Figure 1) based on the presence of a C5-cytosine-specific DNA methylase domain (Supplementary Data Sheet S9). Because Type II restriction endonucleases exhibit extensive sequence diversity and lack universally conserved functional motifs, no specific PFAM signatures could be used for their reliable identification. Instead, their annotation was primarily supported by genomic context and proximity to their cognate methyltransferase genes. Each methyltransferase gene was located adjacent to a restriction-associated protein (End1, End2 and End3), with partial overlap between the *mhy3* and *end3* genes (Figure 1).

To assess the evolutionary relationships and potential cognate associations of these proteins, separate phylogenetic analyses were performed for methyltransferases and restriction-associated proteins using representative Type II restriction-modification families (Figure 3A). In all three loci, the phylogenetic clustering of each restriction-associated protein was consistent with that of its adjacent methyltransferase. End1 and Mhy1 clustered with proteins related to the MAG4270 restriction enzyme and methyltransferase from *M. agalactiae*, End2 and Mhy2 clustered with UPV_230 and UPV_229 from *Ureaplasma parvum*, and End3 and Mhy3 clustered with Sau3AIR- and Sau3AIM-related proteins from *Staphylococcus aureus*. These phylogenetic patterns support the functional association between neighboring restriction-associated proteins and methyltransferases within each genomic locus.

**Figure 3:**
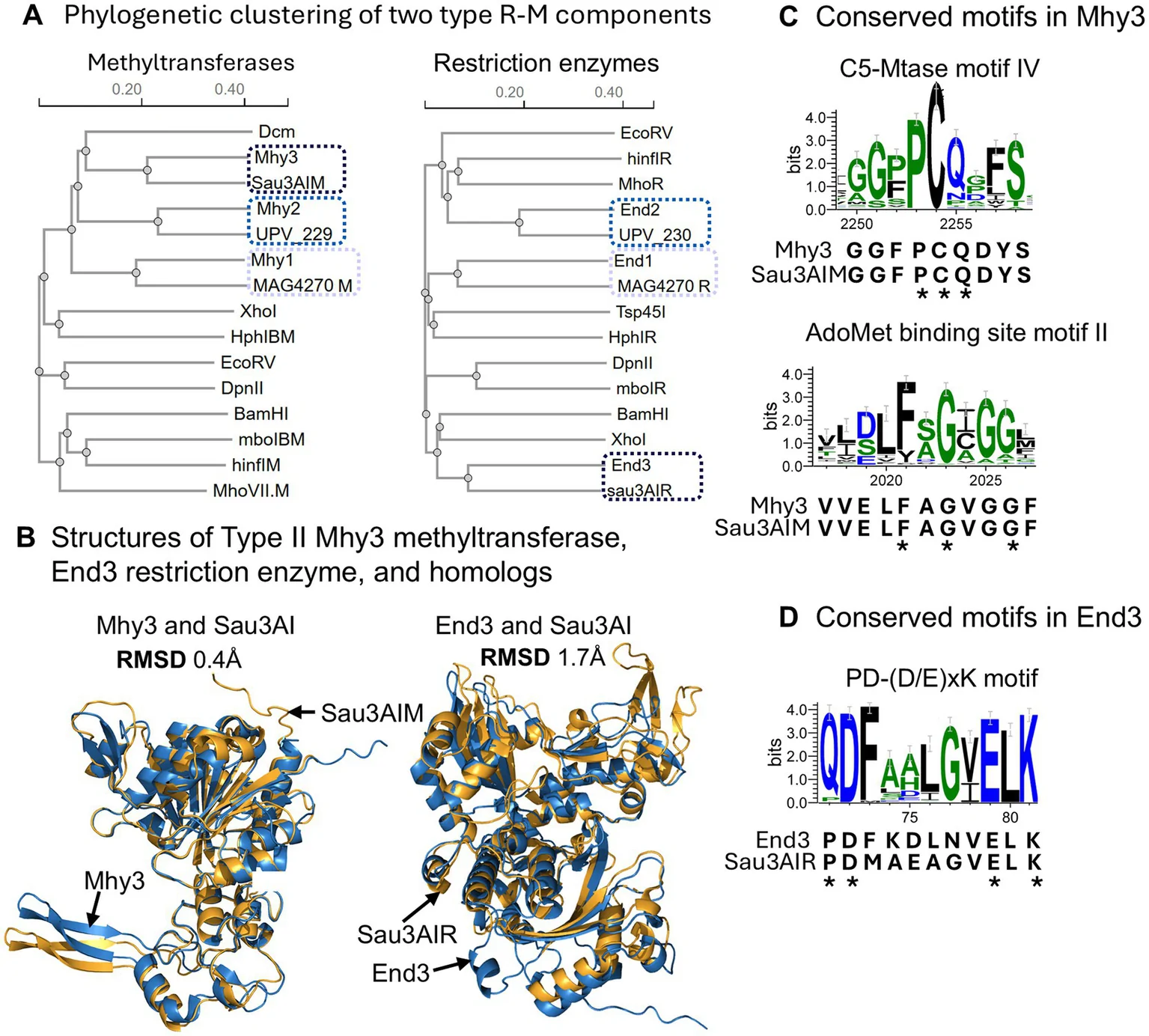
Structural and phylogenetic characterization of Type II R-M system components in *M. hyorhinis* SK76. **(A)** Maximum-likelihood phylogenetic trees showing the relationships of Type II methyltransferases and restriction enzymes from *M. hyorhinis* SK76 relative to well-characterized restriction enzymes from other bacteria. Colored boxes highlight SK76 proteins (End1-End3 and Mhy1-Mhy3) to facilitate comparison. Trees were generated using Clustal alignments (default settings) to determine the phylogenetic family of each endonuclease and methyltransferase. **(B)** Structural superposition of Mhy3 and End3 of *M. hyorhinis* (blue) with representative members of the Sau3AI family (orange), showing overall fold conservation. **(C,D)** Sequence logos derived from alignments of Mhy3 with C5 methyltransferases and End3 with Sau3AI-family restriction endonucleases, highlighting conserved motifs. Asterisks indicate conserved residues associated with cofactor binding or catalytic activity.

Proteomic analysis revealed marked differences in expression among the three systems (Table 2). Mhy3 was confidently detected at high abundance, together with its associated restriction protein End3. In contrast, neither Mhy1 nor Mhy2 was detected despite the presence of proteotypic peptides in their sequences. End1 was not detected, whereas End2 was detected only at the threshold of confidence and was supported by a limited number of proteotypic peptides.

Structural analysis was therefore focused on the Mhy3-End3 pair. AlphaFold 3 models showed high structural similarity to the *S. aureus* Type II Sau3AI methyltransferase and restriction enzyme, with RMSDs of 0.4 A and 1.7 A, respectively (Figure 3B). Sequence analysis of Mhy3 identified the conserved PCQ catalytic motif characteristic of C5 cytosine methyltransferases and the FxGxG motif involved in S-adenosyl-L-methionine binding (Figure 3C; Supplementary Data Sheet S8). End3 retained the PD-(D/E)XK nuclease motif (Figure 3D).

Given the lack of confident detectable expression of the Mhy1-End1 and Mhy2-End2 systems, structural analysis was therefore focused on the Mhy3-End3 pair. AlphaFold 3 models showed high structural similarity to the *S. aureus* Type II Sau3AI methyltransferase and restriction enzyme (Uniprot IDs P16668 and P16667), with (RMSD of 0.4 Å and 1.7 Å, respectively) (Figure 3B). Sequence alignment of Mhy3 identified the conserved PCQ catalytic motif characteristic of C5 cytosine methyltransferases and the FxGxG motif involved in S-adenosyl-L-methionine binding (Jeltsch, 2002) (Figure 3C; Supplementary Data Sheet S9). End3 retained the PD-(D/E)XK nuclease motif, consistent with a catalytically competent Type II restriction endonuclease and supporting its predicted functionality (Figure 3D), although its restriction activity remains to be directly demonstrated.

### *In silico* characterization and proteomic evidence for type III R-M components in *Mycoplasma hyorhinis* SK76

The Type III R-M system is organized as a compact genomic locus containing multiple Mod-related genes clustered in tandem (*mod1*, *mod2B*, *mod2A*, *mod3B* and *mod3A*) adjacent to a predicted Res restriction subunit (*res1*) (Figure 1A). Among the Mod sequences, only Mod1 retained both the glycine-rich FxGxG AdoMet-binding motif and the catalytic DPPY motif characteristic of active Type III methyltransferases (Figure 4A) (Ma et al., 2016). The *mod1* gene contains an intragenic 11-repeat AG-rich short sequence repeat (SSR) (Figure 1; Supplementary Data Sheet S1). SSRs can undergo slipped-strand mispairing during DNA replication, generating repeat-length variants that can alter the reading frame. This process, known as phase variation, generates stochastic genetic variation within bacterial populations (Moxon et al., 2006).

**Figure 4:**
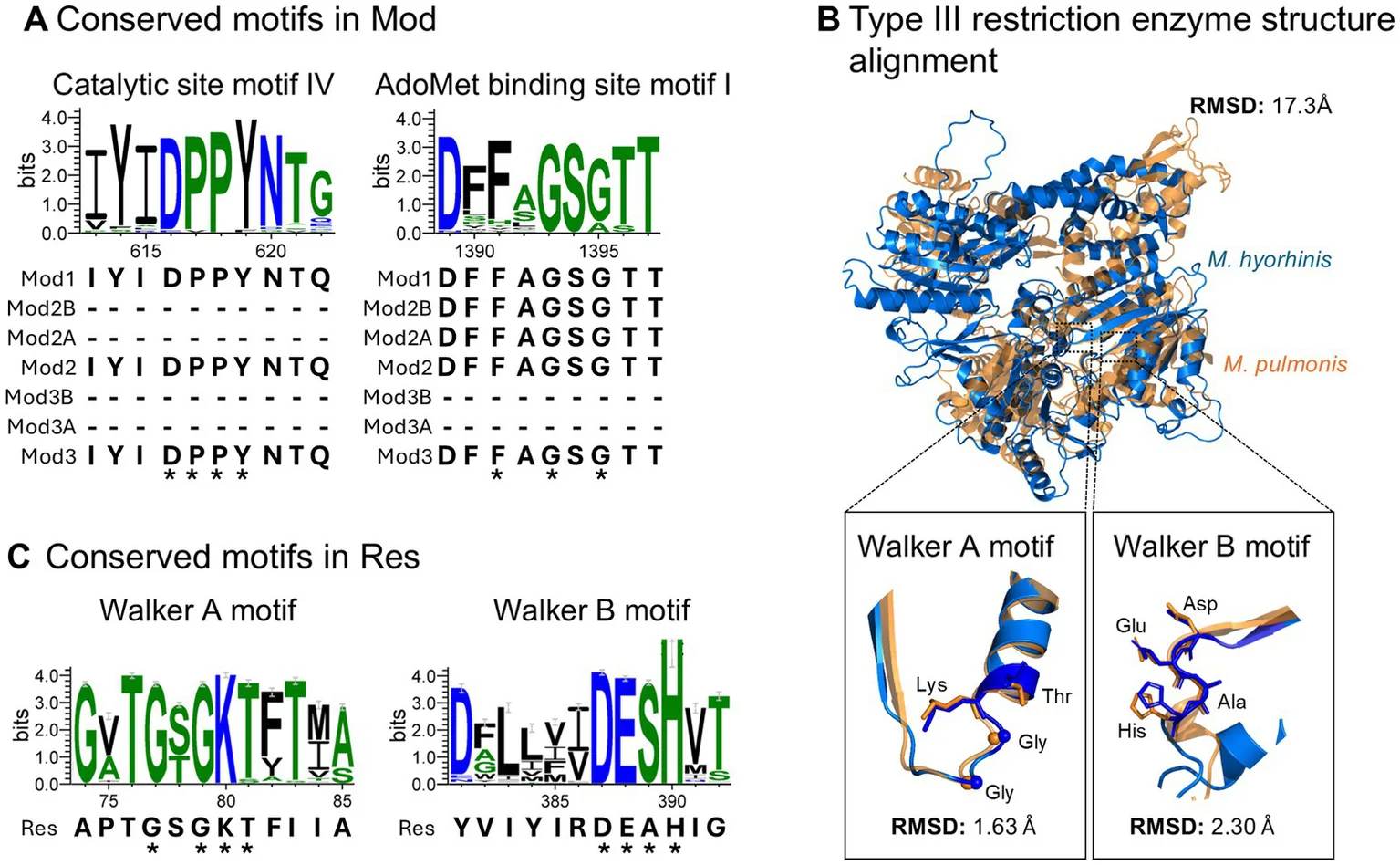
Structural and sequence analysis of Type III R-M system components in *M. hyorhinis* SK76. **(A)** Sequence logos from the alignment of *M. hyorhinis* Mod proteins with reviewed Type III Mod methyltransferases, highlighting conserved catalytic and AdoMet-binding motifs. Alignments of Mod1, Mod2A, Mod2B, Mod3A and Mod3B variants highlight the positions of key residues required for methyltransferase activity. Asterisks indicate residues essential for catalytic function. Mod2 and Mod3 indicate potential full-length proteins generated via SSR-mediated frameshifts. **(B)** Predicted structure alignment of Res *M. hyorhinis* SK76 Res (blue) is superimposed on *M. pulmonis* MYPU3950 (orange) to show conserved fold organization. Domains are highlighted with dashed squares corresponding to Walker A and B motifs. **(C)** Sequence logos from the alignment of *M. hyorhinis* Res1 with reviewed Res proteins, highlighting Walker A and B motifs. Key residues for ATP-dependent DNA translocation are marked with asterisks.

Comparative analysis of RefSeq and GenBank annotations revealed discrepancies in the predicted start codons of Mod2A and Mod3A. RefSeq predicts N-terminal extensions initiated at non-canonical ATC codons, whereas the GenBank annotations correspond to more biologically plausible start sites. According to the GenBank annotation, Mod2A and Mod3A are truncated proteins that retain the FxGxG AdoMet-binding motif but lack the catalytic DPPY motif found in Mod2B and Mod3B, respectively (Figure 4A), indicating that they are unlikely to function as independent methyltransferases. SSRs were identified between *mod2A*-*mod2B* (Mod2) and *mod3A*-*mod3B* (Mod3) (Figure 1A). Sequence reconstruction showed that a two-base deletion and a one-base deletion, respectively, would restore continuous in-frame mod2 and mod3 open reading frames encoding proteins containing both conserved motifs (Supplementary Data Sheet S11; Figure 4A).

Phylogenetic analysis placed Mod1 with full-length Mod methyltransferases from other Mycoplasma species, whereas Mod2 and Mod3 formed closely related but shorter divergent branches within the same lineage (Supplementary Figure S1). Res1 displays structural similarity to the well-characterized Type III restriction enzyme from *M. pulmonis* (Dybvig et al., 2007) (Figure 4B). It also preserves the canonical domain architecture, comprising the Type III Res domain along with helicase-associated domains (Supplementary Data Sheet S9). Sequence analysis further revealed conserved Walker A and Walker B ATPase motifs (Figure 4C), consistent with the ATP-dependent DNA translocation activity typical of Type III Res proteins (Mackeldanz et al., 2013).

Proteomic analysis identified unique peptides assigned to Mod1, Mod2B, and Mod3B, whereas the high sequence similarity among Mod variants prevented confident detection of Mod2A, Mod3A or putative SSR-restored full-length products (Table 2; Supplementary Data Sheet S4). Mod1 and Mod2B showed similar protein abundance, whereas Mod3B was less abundant. The truncated organization of the *mod2*-*mod3* region was conserved across multiple *M. hyorhinis* strains (Supplementary Figure S2). Res1 was detected at an abundance like Mod1.

### Type I, II and III R-M system genes are broadly conserved and largely essential across *M. hyorhinis* strains

Conservation of the predicted Type I, II and III R-M genes was analyzed across 22 *M. hyorhinis* strains using SK76 as the reference (Figure 5A; Supplementary Data Sheet S6; Supplementary Figure S2). Homologs of Type I HsdM, HsdR, and HsdS subunits were identified in most strains, with specificity subunits generally showing >70% sequence identity. The three Type II cytosine methyltransferases (Mhy1-Mhy3) were highly conserved across strains, while the corresponding restriction endonucleases were mostly preserved; End1 was absent in JF5820 and its derivatives. IVB14_OD_0817 contains End2 gene but lacks a Mhy2 homolog. The Type III R-M system was largely conserved, with genes absent in only 4 of the 22 strains analyzed. While IVB16_OD_0575 carries a *res* gene, its activity would require a Mod subunit due to the interdependence of Type III components for restriction activity.

**Figure 5:**
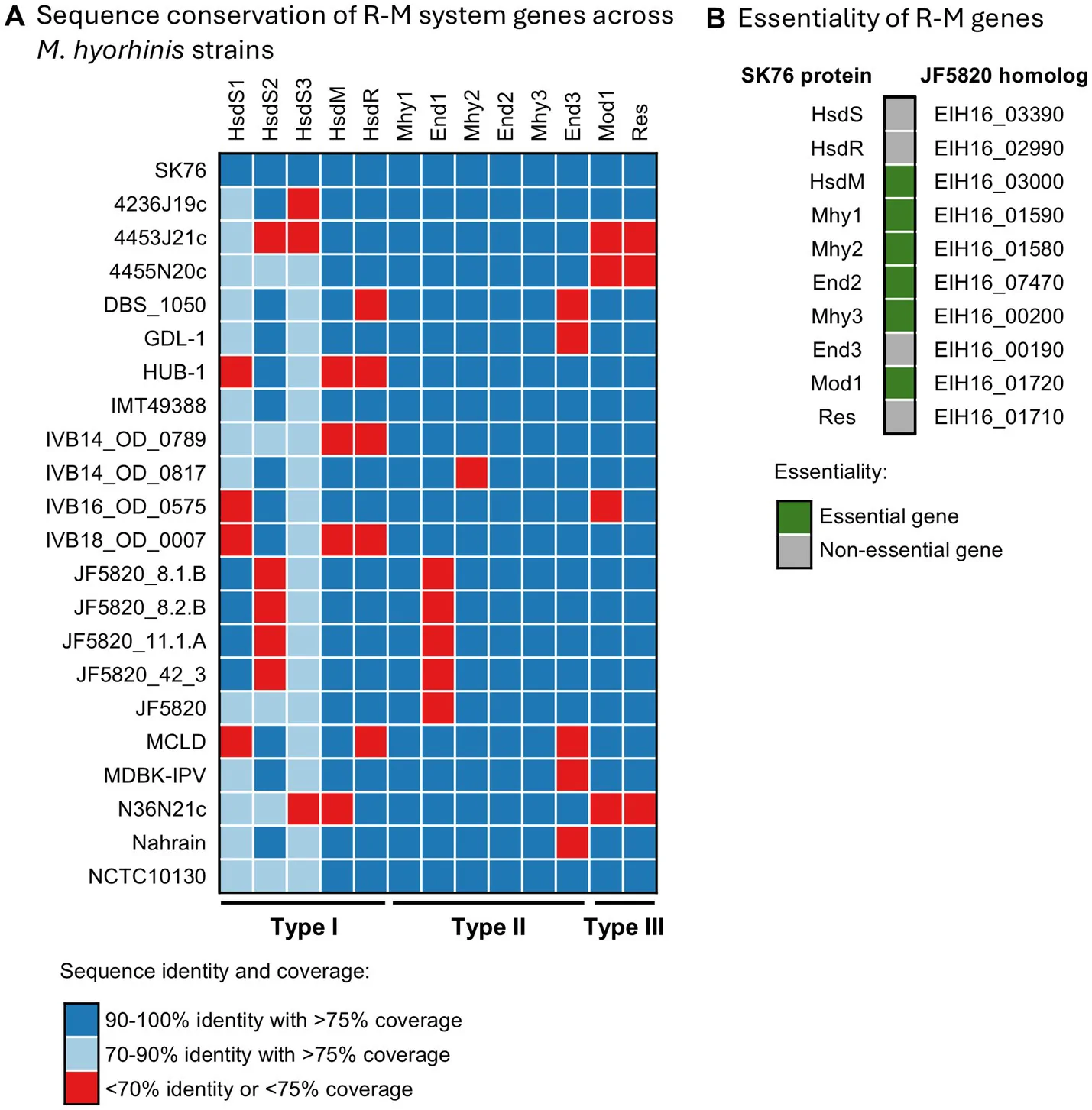
Conservation and essentiality of R-M system genes across *M. hyorhinis* strains. **(A)** Sequence conservation of R-M system genes across multiple *M. hyorhinis* strains. Color intensity reflects both sequence identity and alignment coverage: dark blue indicates >90% identity with >75% coverage, light blue indicates 70–90% identity with >75% coverage, and red indicates <70% identity or <75% coverage. This representation highlights both the presence of homologous genes and their degree of sequence conservation. **(B)** Essentiality of R-M system genes in SK76 inferred from homologs in *M. hyorhinis* JF5820 (Trueeb et al., 2019). Genes are classified as essential (green) or non-essential (grey), showing which components are likely required for viability based on prior functional studies.

Essentiality data from *M. hyorhinis* JF5820 (Trueeb et al., 2019) were mapped onto the corresponding SK76 R-M genes (Figure 5B). All methyltransferases across the three R-M types were classified as essential. End1 and End3 were classified as non-essential, whereas End2 was classified as essential. Despite this, the proteomic analysis detected End2 only at very low levels in the SK76 strain. Within the Type III system, Mod1 was classified as essential and Res1 as non-essential (Figure 5B). The high conservation of Mod1 across strains, combined with its essentiality, highlights its central role in maintaining the Type III methylation system in *M. hyorhinis* SK76. Among all strains analyzed, only N36N21c lacks two methyltransferases of different R-M system types, whereas all other strains are missing at most one or none of the three R-M system types.

### *M. hyorhinis* SK76 has a multi-layered DNA methylation landscape

Adenine methylation was reported only in *M. hyorhinis* ATCC 17981 strain (Razin and Razin, 1980). To confirm the presence of 6 mA, genomic DNA from *M. hyorhinis* SK76 was analyzed by dot-blot using an anti-6 mA antibody. A strong signal was detected in *M. hyorhinis* DNA, exceeding that observed in *E. coli* DH5a wild-type DNA, whereas no signal was detected in DNA from *E. coli dam^−^/dcm^−^* strain (Figure 6A; Supplementary Figure S3).

**Figure 6:**
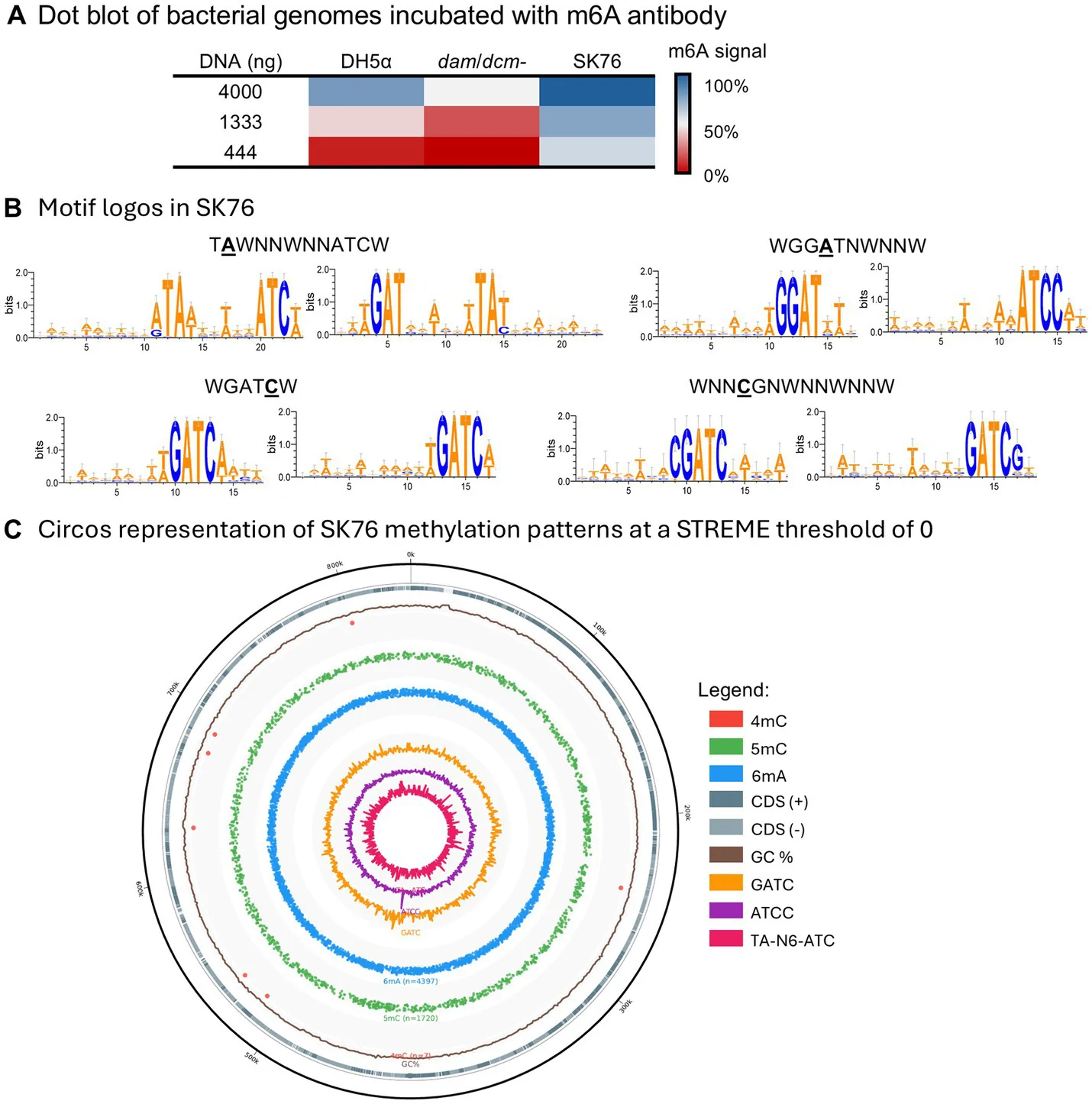
DNA methylation analysis of *M. hyorhinis* SK76. **(A)** Dot blot analysis of bacterial genomic DNA incubated with an 6 mA-specific antibody, showing relative methylation levels in DH5α, *E. coli dam^−^/dcm*^−^, and *M. hyorhinis* SK76 strains. DNA amounts are indicated. **(B)** Sequence logos representing enriched motifs associated with 6 mA and 5mC modifications in SK76, derived from Nanopore methylome profiling. Methylated nucleotide is bold and underlined. **(C)** Circos plot illustrating genome-wide methylation patterns in SK76.

Genome-wide 6 mA, 5mC and 4mC modifications were subsequently analyzed by Oxford Nanopore sequencing and MicrobeMod (Galeone et al., 2025) using STREME thresholds of 0, 0.5 and 0.9 to refine consensus sequence detection (Table 3; raw outputs in Supplementary Data Sheet S3). At a STREME threshold of 0, multiple candidate motifs were detected for all three modification types, although these showed low average per-site methylation levels (<0.25) (Supplementary Data Sheet S3). However, at moderate stringency (STREME threshold of 0.5), two predominant 6 mA-associated motifs and one with very low coverage were identified. The first one corresponds to a non-palindromic sequence, 5′-WGG**AT**NWNNW-3′, detected at 2.333 genomic sites with 77.2% methylation coverage. The modified nucleotide corresponds to the adenine at position 4 within the consensus, consistent with N6-methyladenine modification. Signal was also detected at position 5 with approximately 50% distribution, reflecting methylation of the complementary adenine in the opposite strand orientation. No global strand bias was observed in the genome-wide modification calls. A secondary bipartite motif, 5′-T**A**WNNWNNA**T**CW-3′, was also detected with low frequency and coverage. In this case, positions 2 and the complementary A to T at position 10 have a signal of 62.6 and 30.4%, respectively. The third one with very low coverage had the motif W**GATC**W, with the A methylated mainly in that strand (Table 3).

**Table 3:** Genome-wide DNA methylation motifs detected in *M. hyorhinis* SK76.

Modification	Motif	Pattern	Genome sites	Coverage	Modified % (strand)
0.5 STREME threshold					
6 mA	5’-W**GGAT**NWNNW-3′ 3’-W**CCTA**NWNNW-5’	Non-palindromic	2,333	0.772	+: 47.5 −: 50.7
6 mA	5’-**TA**WNNWNN**ATC**W-3′ 3′- **AT**WNNWNN**TAG**W -5’	Bipartite	890	0.472	+: 62.6 −: 30.4
6 mA	5′- **AAGATC**-3′ 3′- **TTCTAG**-5’	Non-palindromic	637	0.088	+: 89.5
5mC	5′- W**GATC**W-3′ 3′- W**CTAG**W -5’	Palindromic	2,320	0.901	+: 50 −: 50
5mC	5′- WNN**CG**NW**N**NWNNW -3′ 3′- WNN**GC**NWNNWNNW -5’	Palindromic	4,200	0.031	+: 30.5 −: 39.0
4mC	5′- M**CATCC**NW -3′ 3′- M**GTAGG**NW -5’	Non-palindromic	123	0.016	−: 100
0.9 STREME threshold					
6 mA	5’-WNNWNNW**GGAT**NW-3′ 3′- WNNWNNW**CCTA**NW -5’	Non-palindromic	1956	0.774	+: 46.4 −: 49.6
5mC	5’-WNNWW**GATC**WWNNW-3′ 3′- WNNWW**CTAG**WWNNW-5’	Palindromic	824	0.932	+: 49.8 −: 49.8

Motif orientation was standardized for comparative purposes; reverse complements of STREME-derived motifs are presented for each motif. Core sequences are shown in bold, and methylated nucleotides are underlined. Motifs were identified from Nanopore sequencing Data using MicrobeMod with different STREME thresholds (0.5 and 0.9). Columns indicate: Modification, the type of modified base; Motif, the consensus sequence of the methylated site; Pattern, the motif symmetry (palindromic or bipartite); Genome sites, the total number of occurrences of the motif in the genome; Coverage, the fraction of reads covering the site; and Modified % (strand), proportion of DNA molecules on each strand showing methylation at the specific site, reflecting the frequency and penetrance of modification in the genome. The extended table is found in Supplementary Data Sheet S3.

For 5mC, the predominant motif was the palindromic 5′-W**G**AT**C**W-3′ sequence (2,320 sites; 90.1% coverage) (Table 3). An additional 5′-WNN**CG**NW**N**NWNNW-3′ motif was detected at substantially lower coverage (Table 3). A 4mC **C**ATCC centered motif but also at very low frequency (Supplementary Data Sheet S3).

At a STREME threshold of 0.9, the predominant 6 mA signal retained the GGAT-centered core and was represented as 5′-WNNWNNWGG**AT**NW-3′ (1,956 sites; 77.4% coverage), which corresponds to the same GG**AT**-centered sequence identified at lower stringency thresholds. The apparent shift from GG**AT** to **AT**CC in Supplementary Data Sheet S3 reflects the reverse-complement orientation of the motif, as STREME may report motifs from either DNA strand depending on site representation. Similarly, the principal 5mC signal resolved into an extended palindromic GATC-centered motif (5′-WNNWW**GATC**WWNNW-3′; 824 sites; 93.2% coverage). The bipartite 6 mA-associated motif (TA-N6-ATC configuration) detected at moderate stringency was not recovered at the higher threshold.

Sequence logos of the methylation motifs identified at STREME 0.5 with confident methylation coverage are shown in Figure 6B. Overall, the predominant methylation signals comprised a GATC-centered 5mC motif and a GGAT-centered non-palindromic 6 mA motif, together with a bipartite 6 mA-associated motif detected at moderate stringency.

Methylation signals were broadly distributed across the genome (Figure 6C), with 6 mA more widespread than 5mC. In contrast, only seven 4mC sites were detected genome-wide at STREME 0, providing no evidence of a robust motif-associated 4mC methylation signal in the Nanopore analysis. The predominant 6 mA (GGAT-associated) and 5mC (GATC-centered) motifs were distributed throughout the genome rather than concentrated in specific regions.

### Integrative characterization of active methyltransferase components and putative cognate restriction enzymes

Experimental characterization of restriction-modification systems in *M. hyorhinis* SK76 is constrained by the limited genetic tractability of this species and the lack of robust genome editing tools (Ishag et al., 2017). Furthermore, Type I and Type III R-M systems are heterooligomeric complexes composed of multiple subunits whose functional expression outside the native host has been rarely and laboriously achieved in the literature, in contrast to simpler Type II systems (Roberts et al., 2015). Consequently, genomic organization, conserved catalytic motifs, proteomic evidence, structural modeling and Nanopore-derived methylome profiles were integrated to assign candidate methylation motifs to the R-M components identified in SK76 (Table 4), following similar established practices in bacterial epigenomics (Beaulaurier et al., 2019).

**Table 4:** DNA methyltransferases and putative cognate restriction enzymes in *M. hyorhinis* SK76.

Type	Methyltransferase	Putative centered motif	Restriction endonuclease	Reference
I	HsdM1/HsdS2	**TA**-N6-**ATC**	HsdR1	This study
II	Mhy1	**CG**	End1	Gonzalez et al. (2015)
	Mhy2	R**CATG**Y and **CG**	End2	Gonzalez et al. (2015)
	Mhy3	**GATC** and **CGATCG**	End3	This study and Gonzalez et al. (2015)
III	Mod1	**GGAT**	Res1	This study

Summary of the enzymes responsible for DNA methylation, the primary sequence motifs recognized, and their putative cognate restriction enzymes according to each R-M system type. Core sequences are shown in bold, and methylated nucleotides are underlined. Enzyme associations and motif assignments are inferred from methylome profiling, genomic organization, proteomics, and structural analyses. R (Guanine or Adenine), Y (Cytosine or Thymine).

Type I R-M systems typically recognize bipartite DNA motifs composed of two specific recognition elements separated by a 6–8 bp non-specific spacer (Murray, 2000). Consistent with this architecture, HsdS2 is the most plausible HsdS variant expected to recognize the bipartite 5’-**TA**WNNWNN**ATC**W-3′ motif, based on the structural integrity and proteomic detection of HsdS2 together with the bipartite organization of the detected motif.

Type II methyltransferases generally methylate bases within short recognition motifs, often palindromic, and are capable of catalyzing 5mC, 6 mA or 4mC modifications (Passeri et al., 2024). Mhy3 and the predicted cognate restriction protein End3 were the only methyltransferase-restriction pair robustly detected by proteomics. Consistent with previous reports, Mhy3 targets GATC-containing motifs (Gonzalez et al., 2015), while End3 belongs to the Sau3AI family, which also recognizes GATC (Yahui Liu et al., 2023). Together with the MicrobeMod results, these findings suggest that the secondary methylation motif is better represented as 5′-**CGATCG**-3′ motif compatible with that reported in Table 3 (5′-**CG**NWN**G**-3).

Type III systems predominantly mediate 6 mA methylation, although 4mC-modifying variants have also been reported, and they typically recognize short asymmetric or non-palindromic DNA sequences (Meisel et al., 1995; Iain A. Murray et al., 2021). In SK76, Mod1 was identified as the principal candidate responsible for the 5′-**GGAT**-3′ centered 6 mA signal, supported by the presence of conserved catalytic motifs and its detection in the proteome. Integration of these data with Nanopore methylation profiles confirms that the 5′-**GGAT**-3′ motif is consistent with Mod1 activity.

Overall, this integrative approach enables putative assignment of methylation motifs to their respective R-M components, leveraging genomic, proteomic, and methylome data to map the functional DNA methylation landscape of *M. hyorhinis* SK76 without requiring direct *in vitro* reconstitution. HsdR1, End3 and Res1 were detected by proteomics and retained conserved sequence or structural features associated with restriction activity. However, DNA cleavage activity was not directly tested, and these proteins are therefore considered putative cognate restriction components.

## Discussion

This study provides an integrated view of the R-M landscape in *M. hyorhinis* SK76 by combining comparative genomics, structural modeling, proteomics and genome-wide methylation profiling. Importantly, it illustrates how functional epigenetic systems can be reconstructed in genetically intractable organisms such as some Mollicutes, where classical genetic manipulation is extremely limited, and functional inference must rely on multi-omics integration. Within this framework, SK76 reveals a multilayered epigenetic architecture composed of Type I, Type II, and Type III R-M modules, each showing distinct degrees of conservation, expression, and contribution to the global methylome. Rather than acting as isolated restriction systems, these modules likely contribute to a layered epigenetic architecture in which functional output is modulated by gene retention, protein abundance, and potential phase-variable mechanisms.

The SK76 genome encodes a complete Type I R-M system with a canonical HsdR-HsdM-HsdS organization, consistent with recognition of a bipartite TA-N₆-ATC motif. However, the specific contribution of individual HsdS subunits to DNA recognition cannot be resolved due to the presence of three putative HsdS variants, two of which were identified in the proteomic analysis. Among them, HsdS2 emerges as the most likely contributor to this motif, based on its higher proteomic abundance and the consequent likelihood of preferential incorporation into the limiting HsdM-HsdS scaffold. In addition, structural features of HsdS2, including a conserved dual α-helical organization within its core, more consistent with characterized Type I specificity subunit crystals from other bacteria, such as *M. genitalium* (PDB: 1YDX), *Thermoanaerobacter tengcongensis* (PDB: 3OKG) and *E. coli* Type I systems (PDB: 2Y7H), supporting its potential functional relevance. HsdS1 is missing part of one of the helices of the dual α-helix motif, while HsdS3 completely misses one of the helices. This could suggest that neither is functional despite its expression, and they are in a pseudogenization process. Unlike other Mollicutes where HsdS phase variation is frequently driven by SSRs, SK76 lacks such repeats, indicating a more stable architecture (Dybvig et al., 1998; Dybvig and Yu, 1994). This supports a possible role in both genome defense as well as epigenetic regulation rather than exclusively defensive activity.

SK76 encodes three Type II putative cytosine methyltransferases (Mhy1-Mhy3), although only Mhy3 is supported by proteomic detection and the concurrent detection of End3. Moreover, the observed 5’-WGATCW-3’ 5mC motif is fully consistent with the reported specificity of Mhy3 and its related Sau3AI-like restriction system (Gonzalez et al., 2015). In contrast, Mhy1 (recognizing **CG**) and Mhy2 (recognizing R**CATG**Y and **CG** motifs), previously shown to be active by expression in *E. coli* cells (Gonzalez et al., 2015), were not detectably expressed at the protein level under the conditions tested, and no corresponding methylation signatures were observed. These findings provide no evidence for Mhy1- or Mhy2-dependent methylation in this work, although their expression or methylation at levels below the proteomic or sequencing detection threshold cannot be excluded. Overall, these results highlight that cytosine methylation in SK76 is driven by a single active Type II system, reinforcing the idea that functional R-M activity in *M. hyorhinis* represents a subset of its genomic potential rather than a direct reflection of gene content. An open question concerns the biological significance of End2, which was detected at extremely low abundance by proteomics despite the apparent lack of activity of its cognate methyltransferase. One possibility is that the two peptides assigned to End2 represent a low-confidence identification or originate from contaminating proteins present in the culture medium. Alternatively, End2 may be expressed at basal levels that are sufficient for detection but remain below the threshold required for biological activity, or its nuclease activity may be tightly regulated through post-translational or allosteric mechanisms to prevent self-DNA damage in the absence of cognate methylation. Another possibility is that the Mhy2-End2 locus is undergoing functional degeneration in the SK76 strain, resulting in residual expression of End2 despite the apparent loss of methyltransferase activity. Interestingly, transposon mutagenesis data obtained in the JF5820 strain predicted End2 to be essential, suggesting that the functional relevance of this locus may differ between strains or depend on strain-specific regulatory or genomic contexts. Further experimental work will be required to determine whether End2 retains catalytic activity in SK76 or whether it has acquired an alternative cellular role independent of canonical restriction-modification activity. According to REBASE, RCATGY is also recognized by NspI and NspHI methyltransferases from Nostoc.

Among the Type III candidates identified in SK76, Mod1 is the most plausible enzyme responsible for the observed GGAT-centered 6 mA methylation signal, as it retains the expected conserved catalytic motifs, unlike the other Mod candidates, and was robustly detected in the proteomic dataset. The strand-asymmetric nature of this motif further supports a Type III mechanism and distinguishes it from Type I and II contributions. In contrast, the additional Mod2A, Mod2B, Mod3A and Mod3B variants likely represent a more complex and possibly attenuated regulatory architecture. Although SSRs located between these genes have been proposed to enable frameshift-dependent restoration of full-length proteins, such mechanisms remain speculative in SK76. The organization of the mod2-mod3 region was conserved across multiple *M. hyorhinis* strains, suggesting that this architecture is not unique to SK76. These observations indicate that mod1-mod3 region may represent a phase-variable reservoir with the potential to generate methyltransferase diversity in subpopulations. However, even rare restoration events in a small fraction of the population could potentially generate alternative methylation profiles, contributing to epigenetic heterogeneity and allowing adaptive responses under environmental conditions not captured in our experimental setting. Similar phase-variable strategies have been described in other Mollicutes, including *M. bovis* (Ahmed et al., 2026) and *M. agalactiae* (Dordet-Frisoni et al., 2022), where repeat-mediated frameshifting modulates Type III methyltransferase ON/OFF activity and generates epigenetic heterogeneity. However, the lack of proteomic evidence for fusion products, together with the conservation of truncated architecture across strains, argues against efficient restoration of alternative full-length enzymes under the conditions tested. Within this framework, Mod1 emerges as the most likely active methyltransferase responsible for GGAT methylation in SK76, while additional Mod homologs are more likely to represent evolutionary remnants or conditionally regulated elements with limited activity. Given the known propensity of Type III systems to undergo rapid diversification and phase variation, such architectural complexity likely reflects a balance between functional methylation control and evolutionary instability.

Unlike other Mycoplasma species such as *M. bovis*, where 4mC has been reported alongside 6 mA and 5mC (Blow et al., 2016), in *M. hyorhinis* a candidate 4mC motif (CATCC) was identified at a STREME threshold of 0.5, but supported only by two methylated sites and extremely low methylation coverage (0.016). Therefore, this signal most likely represents background noise rather than a biologically relevant methylation event. Regarding the putative 6 mA motif AAGATC, although also displaying relatively low methylation coverage (0.088), it was supported by 56 methylated sites. Importantly, low methylation coverage alone does not necessarily preclude biological relevance. For example, the previously characterized 5mC motif CGATC, experimentally assigned to Mhy3 by heterologous expression in *E. coli* (Gonzalez et al., 2015), was also detected in our methylome with a similarly low methylation coverage (0.031) despite being supported by 129 methylated sites. Thus, while the evidence for AAGATC is insufficient for confident motif assignment, its presence cannot be completely excluded. If confirmed, this motif could reflect residual or condition-dependent activity of an additional adenine methyltransferase that is inactive or only weakly active under the tested conditions or alternatively represent a secondary methylation specificity of one of the methyltransferases already identified in SK76.

Overall, the epigenetic complexity observed in SK76 is consistent with the remarkable diversity of methylation systems reported across the genus *Mycoplasma*. For example, single-base methylome analyses of *M. pneumoniae* and *M. genitalium* revealed markedly different repertoires of methylated motifs despite their close phylogenetic relationship, demonstrating that epigenetic landscapes can diverge substantially even among related species (Lluch-Senar et al., 2013). In contrast, *M. gallisepticum* displays a highly simplified and constrained methylome, with only a single detectable motif (Semashko et al., 2022), further illustrating the remarkable diversity of DNA methylation systems across the genus. Comparative genomic analysis across multiple *M. hyorhinis* strains indicates that R-M systems are broadly conserved, with methyltransferases showing higher conservation than restriction enzymes, consistent with a predominant role in DNA methylation-mediated processes rather than restriction alone. This is further supported by the apparent essentiality of key methyltransferases and the dispensability of most restriction components. However, further studies are needed to understand the complete essentiality of these genes, as many examples require multiple gene knockouts. The data support a model in which HsdM, Mhy3 and Mod1 are the principal active methyltransferases under laboratory conditions, while additional enzymes may be regulated by phase variation, differential expression or conditional activation. The absence of obvious pseudogenized components or orphan methyltransferases further suggests that these systems are not undergoing degenerative loss. Instead, their maintenance despite the reduced genomic repertoire of Mollicutes implies that they continue to provide a selective advantage, whether through defense against foreign DNA, epigenetic regulation, host interaction, or a combination of these functions. Interestingly, a broad comparative study of *M. hyorhinis* strains (Obregon-Gutierrez et al., 2025) revealed that certain *hsdM* and *hsdR* subunits are absent in commensal strains, whereas the Type I R-M system is consistently maintained in virulent isolates. In addition, the HsdM methyltransferase has been shown to influence virulence in *M. hominis* (Pobeguts et al., 2024) and Type I R-M systems more broadly are known to modulate virulence in other bacterial species (Seib et al., 2020). This, together with the fact that *hsd* system is the only one that varies in expression at protein level between exponential and stationary phase, seems to support their involvement in virulence.

These observations highlight a broader caveat in the interpretation of *in silico* R-M predictions: genomic features such as SSRs or fragmented ORFs do not necessarily translate into functional enzymatic activity as has been done in this case. Careful integration of proteomic and methylome data is therefore essential to avoid over-interpretation of potential R-M complexity in streamlined genomes such as those of Mollicutes.

## Data Availability

The original contributions presented in the study are publicly available. The raw Oxford Nanopore sequencing data generated in this study have been deposited in the European Nucleotide Archive (ENA) under Project accession number PRJEB122860. The mass spectrometry proteomics data have been deposited to the ProteomeXchange Consortium via the PRIDE (Perez-Riverol et al., 2025) partner repository with the dataset identifier PXD080153.

## Footnotes

1
https://sciencecodons.com/tools/palindromic-sequence-finder/

2
https://itol.embl.de/
