# Supplementary material for: Integrative multiomic characterization of the methylome and restriction-modification repertoire of Mycoplasma hyorhinis SK76
Source: Front Microbiol. 2026 Oct 22;17:1950185. doi: 10.3389/fmicb.2026.1950185 (PMC13736764; doi:10.3389/fmicb.2026.1950185)
Supplement: Supplementary file 2 [file Data_Sheet_10.DOCX]

Supplementary Material

**Supplementary Data S10**. Residue ranges defining the TRD1, central, and TRD2 regions of Type I HsdS proteins. Residues were assigned based on the predicted structures from AlphaFold3.

| **Protein** | **TRD1** | **Central** | **TRD2** |
| --- | --- | --- | --- |
| HsdS1 | 5-104 | 105-130 | 131-304 |
| HsdS2 | 16-176 | 177-206 | 207-368 |
| HsdS3 | 21-179 | 180-209 | 210-328 |
