# Supplementary material for: Integrative multiomic characterization of the methylome and restriction-modification repertoire of Mycoplasma hyorhinis SK76
Source: Front Microbiol. 2026 Oct 22;17:1950185. doi: 10.3389/fmicb.2026.1950185 (PMC13736764; doi:10.3389/fmicb.2026.1950185)
Supplement: Supplementary file 3 [file Data_Sheet_11.DOCX]

Supplementary Material

**Supplementary Data S11**. **DNA and protein sequences of Type III Mod variants**. For each Mod protein, the corresponding genomic DNA sequence is shown along with the protein sequence annotated in RefSeq or GenBank. Catalytic motifs described throughout the manuscript (DPPY and FxGxG) are underlined. Nucleotides and residues in blue indicate sequence elements shared between RefSeq and GenBank annotations, while green highlights represent additional residues specific to RefSeq. Nucleotides highlighted in yellow marks AG repeat regions. Positions shown reflect functional importance, including residues modified or removed to generate the mature protein sequence.

| **In-house annotation** | **DNA sequence** | **Protein Genbank** | **Protein RefSeq** |
| --- | --- | --- | --- |
| Mod1 | **ATG**AACAAAAAAGAAGAACTTTTGAAAGATCATTTAAACGAATTAGAACAAATTTCTAAATCATCTTTAAATGAAAATCAAAAAGAATTAATTAAACTAAATTTAGAAATTTTAAAAAATAATGACAAAATTTCTATAGATGACTTTGAACAAATTTGAAGATTTTTAAAACAAAGAGTAGCAACTGGGTTTGTTTTTGACGAAGCACCAGAAATTAATAATCAAAGCATCGCAATACTTGAAAAAGACGAAAAATTATCTTTTTCTTCTTCAAAAATGAAGGATAATTTACAAAATTCTTTAATCATTGGAGAAAATTATGATGTTTTAAAGAACCTTATAGGAGTAGAGAGAGAGAGAGAGAGAGAGGATTCTAACTTCGATTTAATCTACATTGACCCTCCTTATAACACACAAAAGACATCAGATGATGGAAATAATTTAACTGATGATGAAATAACTGCTGATAAATTTATATATCGGGACAAATTTTCAAGAACAGGATGATTAAATCTTTTAAACGAAAGATTAAAATTAGCAAAACAACTTCTAAAAGAAGACGGAGTCATCTTTGTATCCATCGATGATAATGAACAAGCTTATTTAAAAGTTTTAATGGATGAAATTTTTGGTGAAGAAAATTTTGTTGCGAATTTAATTTGAATGAAAAAAACAGGTCCTAGTGGAAATACAAGTTCTCAATATTCTATAGATACAATAACTGAATATATTTTGTGTTATGCTAAAAATAAAAACAAAAAAACTTTTAATTACTTAAAACATAATGAACAATCTTTCTCTGATTCAGGATTTGATTTAAAAGATGAGTTCTTTGACGAAAGAGGTTATTACAGATTAGTGGATTTACACCATGGATCAAGTGCATCTACTTTTAAATATTTAGAAAGTCTTGATTACGAAATAGAAGCTCCAGATGGTACTTTTTTTAAGTTGTTTACCAATATAAAAAAACCCAAATCTGCGTGTTATACTTGATCATATAAAACATATTTAGAAGGTAAAAAACAAGGATTTATTCAGGTAATAAAAAATAACGAAGGATTTTGAGTAGCAAAAAGAAAACAATATCAATTTGTAAAATTTAATCCAAAAACTTTTGAAATAGAAACTCAAGTAGCAGGTCGAAGATTTAATAATGTAATTGATAAAAATTACAATAGATTAAGTAATGGTTTTTACACAACAAAATCAGCTAATGAAATAACTGAAGTTTTATTTGATAAAACGGCATTTAATTTTGCGAAACCTGTTGGTTTGATGAAATATTTAATTAATTTATTTTCTGATAAAAAAGATATGAGAGTTTTAGATTTCTTTGCAGGTTCAGGTACAACTGGACAAGCGGTTTTAGAACTTAATAAAGAAGATGGCGGAAATCGTTCTTTTGTTTTAGTAACTAATAACGAAAATAATATTGGTCAAAACGTAACATATGAAAGACTTTATAGAATTAATAAAGGACAAGGCACAAAAGGTCAAAAAGACTTTGAATGAATCAAGAAAAATGAAGCATTTGATACAAGTTTAAATGTATTTTGATCTAAAACTTACAACACTTCTTTGTTAAATAACAATATAACCAATCAAGAATTAAAAGATAAATTTTTAAAACTTTTAAAAGATTTTGGAATCAATAAAGATAAAGAAAAATTTGATGATTCTGTATTAAAAACTCTTTCTTCTTTAAGACCATATAAAGAAGAAAATGAAGAAAAATAA | AFX74131  MNKKEELLKDHLNELEQISKSSLNENQKELIKLNLEILKNNDKISIDDFEQIWRFLKQRVATGFVFDEAPEINNQSIAILEKDEKLSFSSSKMKDNLQNSLIIGENYDVLKNLIGVEREREREDSNFDLIYIDPPYNTQKTSDDGNNLTDDEITADKFIYRDKFSRTGWLNLLNERLKLAKQLLKEDGVIFVSIDDNEQAYLKVLMDEIFGEENFVANLIWMKKTGPSGNTSSQYSIDTITEYILCYAKNKNKKTFNYLKHNEQSFSDSGFDLKDEFFDERGYYRLVDLHHGSSASTFKYLESLDYEIEAPDGTFFKLFTNIKKPKSACYTWSYKTYLEGKKQGFIQVIKNNEGFWVAKRKQYQFVKFNPKTFEIETQVAGRRFNNVIDKNYNRLSNGFYTTKSANEITEVLFDKTAFNFAKPVGLMKYLINLFSDKKDMRVLDFFAGSGTTGQAVLELNKEDGGNRSFVLVTNNENNIGQNVTYERLYRINKGQGTKGQKDFEWIKKNEAFDTSLNVFWSKTYNTSLLNNNITNQELKDKFLKLLKDFGINKDKEKFDDSVLKTLSSLRPYKEENEEK | WP_015084066  MNKKEELLKDHLNELEQISKSSLNENQKELIKLNLEILKNNDKISIDDFEQIWRFLKQRVATGFVFDEAPEINNQSIAILEKDEKLSFSSSKMKDNLQNSLIIGENYDVLKNLIGVEREREREDSNFDLIYIDPPYNTQKTSDDGNNLTDDEITADKFIYRDKFSRTGWLNLLNERLKLAKQLLKEDGVIFVSIDDNEQAYLKVLMDEIFGEENFVANLIWMKKTGPSGNTSSQYSIDTITEYILCYAKNKNKKTFNYLKHNEQSFSDSGFDLKDEFFDERGYYRLVDLHHGSSASTFKYLESLDYEIEAPDGTFFKLFTNIKKPKSACYTWSYKTYLEGKKQGFIQVIKNNEGFWVAKRKQYQFVKFNPKTFEIETQVAGRRFNNVIDKNYNRLSNGFYTTKSANEITEVLFDKTAFNFAKPVGLMKYLINLFSDKKDMRVLDFFAGSGTTGQAVLELNKEDGGNRSFVLVTNNENNIGQNVTYERLYRINKGQGTKGQKDFEWIKKNEAFDTSLNVFWSKTYNTSLLNNNITNQELKDKFLKLLKDFGINKDKEKFDDSVLKTLSSLRPYKEENEEK |
| Mod2B | **ATC**TACATTGACCCTCCTTATAATACACAAAAGACATCTGATGATGGAAATAATTTAACTGATGATGAAATAACTGCTGATAAATTTATATATCGGGATAAATTTTCAAGAACAGGATGATTAAACCTTTTAAACGAAAGATTAAAATTAGCAAAACAACTTCTAAAAGAAGATGGAGTTATATTTGTTTCAATTGACGATAATGAACAAGCTTATTTAAAAGTGTTA**ATG**GATGAAATTTTTGGTGAAGAAAATTTCGTTGCTAATATCGTTTGAAGGAAATCATCAATTGGTTCTCATAAAGATATAAAAGATATCAAAACTGTCAATGTTGTAAATGAATACATAGTAACCTATGCTAAAAAGAAAACTAAATTAAGATTTGATTATGTAAGACATTCTCAAGAAAAGTTAGACAAAGAATATAAGCTTAAAGATGATAATTTTGAACAATATGGTTATTATAGATTAGAAAGACTAGCATATAAAGGATTGGATTATCAAGCTTCTCTAGATTATGAGTTAGAAGCTCCTGATGGAACTAAATTTAGAATTTATCAAAATATTAAAAAACCACAAACAATGTGTTATATATGATCCAAAGAACTATTTGACTATGCGAATTCTTTAAATTTAGTAGAAATCAAAAAAACTCAACAAGGCAACTGAGTTGCTTATAAAAAAGTTTATCAATATGCTAAATTCGATGCAAGAACTAAACAATTTATCTTAGTTGAAAAAGGCGTTCCTTTTACTAATATGATTATTGCTAATCAAAACAATCTACATTTGAATATTTTGACTGAACAAGGTTCTAAAGAAATGACAAGCATTTTCAAAGATAAAATGTTTGACTATCCAAAACCAGTTGAATTAGTAAAGTATTTAATTAAAATGGCATCTAGCAAAAAAGACATTAGAATCCTTGATTTCTTTGCTGGTTCTGGAACTACTGCACAAGCCGTTTTAGAACTCAACAAAGAAGATGGTGGAAGACGTTCTTTTGTTTTAGTAACTAATAATGAAAATAATATTGGTCAAAACGTGACATATGAAAGACTTTATAGAATTAATAAAGGACAAGGCACAAAGGGTCAAAAAGACTTTGAATGAATCAAGAAAAACGAAGCATTTGATACAAGTTTAAATGTATTTTGATCTAACACTTACAATACTTCTTTGTTAAATAACAATATAACCAATAAAGAATTAAAAGATAAATTTTTAAAACTTTTAAAAGATTTCGGAATCAATAAAGATAAAGAAAAATTTGATGATTCTGTACTAAAAACTCTTTCTTCTTTAAGACCATACAAAGAAGAAAATGAAGAAAAATAA | AFX74132.1  MDEIFGEENFVANIVWRKSSIGSHKDIKDIKTVNVVNEYIVTYAKKKTKLRFDYVRHSQEKLDKEYKLKDDNFEQYGYYRLERLAYKGLDYQASLDYELEAPDGTKFRIYQNIKKPQTMCYIWSKELFDYANSLNLVEIKKTQQGNWVAYKKVYQYAKFDARTKQFILVEKGVPFTNMIIANQNNLHLNILTEQGSKEMTSIFKDKMFDYPKPVELVKYLIKMASSKKDIRILDFFAGSGTTAQAVLELNKEDGGRRSFVLVTNNENNIGQNVTYERLYRINKGQGTKGQKDFEWIKKNEAFDTSLNVFWSNTYNTSLLNNNITNKELKDKFLKLLKDFGINKDKEKFDDSVLKTLSSLRPYKEENEEK | WP_420704347  IYIDPPYNTQKTSDDGNNLTDDEITADKFIYRDKFSRTGWLNLLNERLKLAKQLLKEDGVIFVSIDDNEQAYLKVLMDEIFGEENFVANIVWRKSSIGSHKDIKDIKTVNVVNEYIVTYAKKKTKLRFDYVRHSQEKLDKEYKLKDDNFEQYGYYRLERLAYKGLDYQASLDYELEAPDGTKFRIYQNIKKPQTMCYIWSKELFDYANSLNLVEIKKTQQGNWVAYKKVYQYAKFDARTKQFILVEKGVPFTNMIIANQNNLHLNILTEQGSKEMTSIFKDKMFDYPKPVELVKYLIKMASSKKDIRILDFFAGSGTTAQAVLELNKEDGGRRSFVLVTNNENNIGQNVTYERLYRINKGQGTKGQKDFEWIKKNEAFDTSLNVFWSNTYNTSLLNNNITNKELKDKFLKLLKDFGINKDKEKFDDSVLKTLSSLRPYKEENEEK |
| Mod2A | **ATG**AACAAAAAAGAAGAACTTTTAAAAGATCATTTAAAGGAGTTAGAACAAATTTCTAAATCATCTTTAAATGAAAATCAAAAAGAATTAATTAAATTAAATTTAGAGATTTTAAAAAATAATGACAAAATTTCTATAGATGACTTTGAACAAATTTGAAGATTTTTAAAACAAAGAGTAGCAACTGGATTTGTTTTTGATGAAGCACCAGAAATCAATAACCAAAGCATTGCAATACTAGAAAAAGACGAAAAATTATCTTTTTCTTCTTCAAAAATGAAGGATAATTTACAAAATTCTTTAATTATTGGAGAAAATTACGATGTTTTAAAGAACCTTATAGGAGTAGAGAGAGAGAGAGGATGCTAA | AFX74133  MNKKEELLKDHLKELEQISKSSLNENQKELIKLNLEILKNNDKISIDDFEQIWRFLKQRVATGFVFDEAPEINNQSIAILEKDEKLSFSSSKMKDNLQNSLIIGENYDVLKNLIGVERERGC | NA |
| Mod2 | **ATG**AACAAAAAAGAAGAACTTTTAAAAGATCATTTAAAGGAGTTAGAACAAATTTCTAAATCATCTTTAAATGAAAATCAAAAAGAATTAATTAAATTAAATTTAGAGATTTTAAAAAATAATGACAAAATTTCTATAGATGACTTTGAACAAATTTGAAGATTTTTAAAACAAAGAGTAGCAACTGGATTTGTTTTTGATGAAGCACCAGAAATCAATAACCAAAGCATTGCAATACTAGAAAAAGACGAAAAATTATCTTTTTCTTCTTCAAAAATGAAGGATAATTTACAAAATTCTTTAATTATTGGAGAAAATTACGATGTTTTAAAGAACCTTATAGGAGTAGAGAGAGAGAGAG~~G~~ATGCTAACTTCGATTTA**ATC**TACATTGACCCTCCTTATAATACACAAAAGACATCTGATGATGGAAATAATTTAACTGATGATGAAATAACTGCTGATAAATTTATATATCGGGATAAATTTTCAAGAACAGGATGATTAAACCTTTTAAACGAAAGATTAAAATTAGCAAAACAACTTCTAAAAGAAGATGGAGTTATATTTGTTTCAATTGACGATAATGAACAAGCTTATTTAAAAGTGTTA**ATG**GATGAAATTTTTGGTGAAGAAAATTTCGTTGCTAATATCGTTTGAAGGAAATCATCAATTGGTTCTCATAAAGATATAAAAGATATCAAAACTGTCAATGTTGTAAATGAATACATAGTAACCTATGCTAAAAAGAAAACTAAATTAAGATTTGATTATGTAAGACATTCTCAAGAAAAGTTAGACAAAGAATATAAGCTTAAAGATGATAATTTTGAACAATATGGTTATTATAGATTAGAAAGACTAGCATATAAAGGATTGGATTATCAAGCTTCTCTAGATTATGAGTTAGAAGCTCCTGATGGAACTAAATTTAGAATTTATCAAAATATTAAAAAACCACAAACAATGTGTTATATATGATCCAAAGAACTATTTGACTATGCGAATTCTTTAAATTTAGTAGAAATCAAAAAAACTCAACAAGGCAACTGAGTTGCTTATAAAAAAGTTTATCAATATGCTAAATTCGATGCAAGAACTAAACAATTTATCTTAGTTGAAAAAGGCGTTCCTTTTACTAATATGATTATTGCTAATCAAAACAATCTACATTTGAATATTTTGACTGAACAAGGTTCTAAAGAAATGACAAGCATTTTCAAAGATAAAATGTTTGACTATCCAAAACCAGTTGAATTAGTAAAGTATTTAATTAAAATGGCATCTAGCAAAAAAGACATTAGAATCCTTGATTTCTTTGCTGGTTCTGGAACTACTGCACAAGCCGTTTTAGAACTCAACAAAGAAGATGGTGGAAGACGTTCTTTTGTTTTAGTAACTAATAATGAAAATAATATTGGTCAAAACGTGACATATGAAAGACTTTATAGAATTAATAAAGGACAAGGCACAAAGGGTCAAAAAGACTTTGAATGAATCAAGAAAAACGAAGCATTTGATACAAGTTTAAATGTATTTTGATCTAACACTTACAATACTTCTTTGTTAAATAACAATATAACCAATAAAGAATTAAAAGATAAATTTTTAAAACTTTTAAAAGATTTCGGAATCAATAAAGATAAAGAAAAATTTGATGATTCTGTACTAAAAACTCTTTCTTCTTTAAGACCATACAAAGAAGAAAATGAAGAAAAATAA  Removing 1 bp | MNKKEELLKDHLKELEQISKSSLNENQKELIKLNLEILKNNDKISMDDFEQIWSFLKQSVATGFVFDEAPEINNQSIAMLEKDEKLSFSSSKMKDNLQNSLIIGENYDVLKNLMGVESESDANFDLIYIDPPYNTQKTSDDGNNLTDDEMTADKFMYRDKFSSTGWLNLLNESLKLAKQLLKEDGVMFVSIDDNEQAYLKVLMDEIFGEENFVANIVWSKSSIGSHKDMKDIKTVNVVNEYMVTYAKKKTKLSFDYVSHSQEKLDKEYKLKDDNFEQYGYYSLESLAYKGLDYQASLDYELEAPDGTKFSIYQNIKKPQTMCYMWSKELFDYANSLNLVEIKKTQQGNWVAYKKVYQYAKFDASTKQFILVEKGVPFTNMIIANQNNLHLNILTEQGSKEMTSIFKDKMFDYPKPVELVKYLIKMASSKKDISILDFFAGSGTTAQAVLELNKEDGGSRSFVLVTNNENNIGQNVTYESLYSINKGQGTKGQKDFEWIKKNEAFDTSLNVFWSNTYNTSLLNNNMTNKELKDKFLKLLKDFGINKDKEKFDDSVLKTLSSLSPYKEENEEK | |
| Mod3B | **TTA**TGATGTTTTAAAGAACCTTATAGGAGTAGAGAGAGAGAGAGAGAGGATGCTAACTTCGATTTAATCTACATTGACCCTCCTTATAATACACAAAAGACATCAGATGATGGAAATAATTTAACTGATGATCAAATAACTGCTGATAAATTTATATATCGGGACAAATTTTCAAGAACTGGATGATTAAACCTTTTAAACGAAAGATTAAAATTAGCAAAACAACTTCTAAAAGAAGATGGAGTTATATTTGTTTCAATTGACGATAATGAACAAGCTTATTTAAAAGTGTTA**ATG**GATGAAATTTTTGGTGAAGAAAATTTCGTAGTTAATTTAATATGAATAAGTGCTTGAGGTAGTAAACAAGATGCAAAATACTTTTCACAAAATCATGAATATATTTTAGTTTATTCAAAAAATATTGAAAAACTGAAGATTAAAAAAATAAACATCGCGGAAAATTCGGACCAGTGAAAATGAAATGAAAAAATTCAAAAATGAGAGAAAAAATTAGCAATCCTTCAAAAAGGAAGTAATGAAGAAACAATTTTTGATCGAAAGAAAATGGCATACATTATTTGATGAAATGAAAAATTAAATTTATTCAAAACGGATAATAATATCGACTTTAATTTAATTACTGAAGACTTAACAACTGATACAATAATTTATGACTACTCTACAAGTGAACGACAATTATTAATTTCACAGGGCTATGTTCCTATAATTCCAAAAACAGTAAGAAAAAATAAATATGGAAGATGAACAGTTTCTGAACAAACTTTTTTAGAAAAAGTTAAAAATAATGAGATTAAAGTTCAGAAAATTAAAGATTCATATCAAGTTTTTAGATATGATGTTCAAGATAAAATACCAAATTCAAAAAAACAAAATCCTAGAAGTGTTGTTTTTAATTCTACATTAAATAAATTAGTAGAAAACACAGACAATCACCAGCAAATACAAAGGGCTTCTACAAGTGATGGAAGTTTGATGCTTCGATCTATTTTTAATTTTGACTCTTTTTTTGACTTTCCAAAACCAGTTAATCTTATCAAATACATAATTGACTTATTTCCTTCTAAAAACACAAGAGTCCTAGATTTCTTTGCAGGTTCGGGTACAACTGGACAAGCAGTTTTAGAACTAAATAAAGAAGATGGCGGAAGTCGCTCTTTTGTTTTAGTAACTAATAATGAAAATAGTATTGGTCAAAACATAACATATGAAAGACTTTATAGAATTAATAAAGGGCAAGGTACAAAAGGTCAAAAAGACTTTGAATGAATCAAGAAAAATGAAGCATTTGATACAAGTTTAAATGTATTTTGATCTAAAACTTACAACACTTCTTTGTTAAATAACAATATAACCAATCAAGAATTAAAAGATAAATTTTTAAAACTTTTAAAAGATTTTGGAATCAACAAAGATAAAGAAAAATTTGATGATTCTGTATTAAAAACTCTTTCTTCTTTAAGACCATATAAAGAAG | AFX74134  MDEIFGEENFVVNLIWISAWGSKQDAKYFSQNHEYILVYSKNIEKLKIKKINIAENSDQWKWNEKIQKWEKKLAILQKGSNEETIFDRKKMAYIIWWNEKLNLFKTDNNIDFNLITEDLTTDTIIYDYSTSERQLLISQGYVPIIPKTVRKNKYGRWTVSEQTFLEKVKNNEIKVQKIKDSYQVFRYDVQDKIPNSKKQNPRSVVFNSTLNKLVENTDNHQQIQRASTSDGSLMLRSIFNFDSFFDFPKPVNLIKYIIDLFPSKNTRVLDFFAGSGTTGQAVLELNKEDGGSRSFVLVTNNENSIGQNITYERLYRINKGQGTKGQKDFEWIKKNEAFDTSLNVFWSKTYNTSLLNNNITNQELKDKFLKLLKDFGINKDKEKFDDSVLKTLSSLRPYKEE | WP_337972343  LWCFKEPYRSREREREDANFDLIYIDPPYNTQKTSDDGNNLTDDQITADKFIYRDKFSRTGWLNLLNERLKLAKQLLKEDGVIFVSIDDNEQAYLKVLMDEIFGEENFVVNLIWISAWGSKQDAKYFSQNHEYILVYSKNIEKLKIKKINIAENSDQWKWNEKIQKWEKKLAILQKGSNEETIFDRKKMAYIIWWNEKLNLFKTDNNIDFNLITEDLTTDTIIYDYSTSERQLLISQGYVPIIPKTVRKNKYGRWTVSEQTFLEKVKNNEIKVQKIKDSYQVFRYDVQDKIPNSKKQNPRSVVFNSTLNKLVENTDNHQQIQRASTSDGSLMLRSIFNFDSFFDFPKPVNLIKYIIDLFPSKNTRVLDFFAGSGTTGQAVLELNKEDGGSRSFVLVTNNENSIGQNITYERLYRINKGQGTKGQKDFEWIKKNEAFDTSLNVFWSKTYNTSLLNNNITNQELKDKFLKLLKDFGINKDKEKFDDSVLKTLSSLRPYKEE |
| Mod3A | **ATG**AACAAAAAAGAAGAACTTTTAAAAGATCATTTAAAGGAATTAGAGCAAATTTCTAAATCATCTTTAAATGAAAATCAAAAAGAATTAATTAAATTAAACTTAGAAATTTTAAAAAATAATGACAAAATTTCTATAGATGATTTTGAACAAATTTGAAGATTTTTAAAACAAAGAGTAGCAACTGGGTTTGTTTTTGATGAAGCACCAGAAATCAATAATCAAAGCATTGCAATACTTGAAAAAGACGAAAAATTATCTTTTTCTTCTTCAAAAATGAAGGATAATTTACAAAATTCTTTAATTATTGGAGAAAATTATGATGTTTTAAAGAACCTTATAGGAGTAGAGAGAGAGAGAGAGAGGATGCTAACTTCGATTTAA | AFX74135  MNKKEELLKDHLKELEQISKSSLNENQKELIKLNLEILKNNDKISIDDFEQIWRFLKQRVATGFVFDEAPEINNQSIAILEKDEKLSFSSSKMKDNLQNSLIIGENYDVLKNLIGVERERERMLTSI | NA |
| Mod3 | **ATG**AACAAAAAAGAAGAACTTTTAAAAGATCATTTAAAGGAATTAGAGCAAATTTCTAAATCATCTTTAAATGAAAATCAAAAAGAATTAATTAAATTAAACTTAGAAATTTTAAAAAATAATGACAAAATTTCTATAGATGATTTTGAACAAATTTGAAGATTTTTAAAACAAAGAGTAGCAACTGGGTTTGTTTTTGATGAAGCACCAGAAATCAATAATCAAAGCATTGCAATACTTGAAAAAGACGAAAAATTATCTTTTTCTTCTTCAAAAATGAAGGATAATTTACAAAATTCTTTAATTATTGGAGAAAATTATGATGTTTTAAAGAACCTTATAGGAGTAGAGAGAGAGAGAGAGAG~~GA~~TGCTAACTTCGATTTAATCTACATTGACCCTCCTTATAATACACAAAAGACATCAGATGATGGAAATAATTTAACTGATGATCAAATAACTGCTGATAAATTTATATATCGGGACAAATTTTCAAGAACTGGATGATTAAACCTTTTAAACGAAAGATTAAAATTAGCAAAACAACTTCTAAAAGAAGATGGAGTTATATTTGTTTCAATTGACGATAATGAACAAGCTTATTTAAAAGTGTTAATGGATGAAATTTTTGGTGAAGAAAATTTCGTAGTTAATTTAATATGAATAAGTGCTTGAGGTAGTAAACAAGATGCAAAATACTTTTCACAAAATCATGAATATATTTTAGTTTATTCAAAAAATATTGAAAAACTGAAGATTAAAAAAATAAACATCGCGGAAAATTCGGACCAGTGAAAATGAAATGAAAAAATTCAAAAATGAGAGAAAAAATTAGCAATCCTTCAAAAAGGAAGTAATGAAGAAACAATTTTTGATCGAAAGAAAATGGCATACATTATTTGATGAAATGAAAAATTAAATTTATTCAAAACGGATAATAATATCGACTTTAATTTAATTACTGAAGACTTAACAACTGATACAATAATTTATGACTACTCTACAAGTGAACGACAATTATTAATTTCACAGGGCTATGTTCCTATAATTCCAAAAACAGTAAGAAAAAATAAATATGGAAGATGAACAGTTTCTGAACAAACTTTTTTAGAAAAAGTTAAAAATAATGAGATTAAAGTTCAGAAAATTAAAGATTCATATCAAGTTTTTAGATATGATGTTCAAGATAAAATACCAAATTCAAAAAAACAAAATCCTAGAAGTGTTGTTTTTAATTCTACATTAAATAAATTAGTAGAAAACACAGACAATCACCAGCAAATACAAAGGGCTTCTACAAGTGATGGAAGTTTGATGCTTCGATCTATTTTTAATTTTGACTCTTTTTTTGACTTTCCAAAACCAGTTAATCTTATCAAATACATAATTGACTTATTTCCTTCTAAAAACACAAGAGTCCTAGATTTCTTTGCAGGTTCGGGTACAACTGGACAAGCAGTTTTAGAACTAAATAAAGAAGATGGCGGAAGTCGCTCTTTTGTTTTAGTAACTAATAATGAAAATAGTATTGGTCAAAACATAACATATGAAAGACTTTATAGAATTAATAAAGGGCAAGGTACAAAAGGTCAAAAAGACTTTGAATGAATCAAGAAAAATGAAGCATTTGATACAAGTTTAAATGTATTTTGATCTAAAACTTACAACACTTCTTTGTTAAATAACAATATAACCAATCAAGAATTAAAAGATAAATTTTTAAAACTTTTAAAAGATTTTGGAATCAACAAAGATAAAGAAAAATTTGATGATTCTGTATTAAAAACTCTTTCTTCTTTAAGACCATATAAAGAAG  Removing 2 bp | MNKKEELLKDHLKELEQISKSSLNENQKELIKLNLEILKNNDKISMDDFEQIWSFLKQSVATGFVFDEAPEINNQSIAMLEKDEKLSFSSSKMKDNLQNSLIIGENYDVLKNLMGVESESESANFDLIYIDPPYNTQKTSDDGNNLTDDQMTADKFMYRDKFSSTGWLNLLNESLKLAKQLLKEDGVMFVSIDDNEQAYLKVLMDEIFGEENFVVNLMWMSAWGSKQDAKYFSQNHEYILVYSKNIEKLKIKKMNIAENSDQWKWNEKIQKWEKKLAILQKGSNEETIFDRKKMAYIIWWNEKLNLFKTDNNIDFNLITEDLTTDTMIYDYSTSERQLLISQGYVPMIPKTVSKNKYGSWTVSEQTFLEKVKNNEIKVQKIKDSYQVFSYDVQDKMPNSKKQNPSSVVFNSTLNKLVENTDNHQQMQSASTSDGSLMLRSIFNFDSFFDFPKPVNLIKYMIDLFPSKNTSVLDFFAGSGTTGQAVLELNKEDGGSRSFVLVTNNENSIGQNMTYESLYSINKGQGTKGQKDFEWIKKNEAFDTSLNVFWSKTYNTSLLNNNMTNQELKDKFLKLLKDFGINKDKEKFDDSVLKTLSSLSPYKE | |
