# Supplementary material for: Integrative multiomic characterization of the methylome and restriction-modification repertoire of Mycoplasma hyorhinis SK76
Source: Front Microbiol. 2026 Oct 22;17:1950185. doi: 10.3389/fmicb.2026.1950185 (PMC13736764; doi:10.3389/fmicb.2026.1950185)
Supplement: Supplementary file 4 [file Data_Sheet_2.ZIP › Supplementary_Data_S2.docx]

Supplementary Material

**Supplementary Data S10**. **Summary of the structural models used throughout this study.** The table lists the models employed in the analyses, together with their corresponding file names (available in this supplementary dataset), prediction confidence (pTM), interface predicted template modeling (iPTM), manuscript figure references, structural alignment references, and the random seed used for model generation.

| **Model** | **File name** | **Confidence** | **Manuscript figure reference** | **Alignment reference** | **Seed** |
| --- | --- | --- | --- | --- | --- |
| HsdS1 | HsdS1_larger | pTM: 0.76 | Figure 2.A | NA | 858116784 |
| HsdS2 | AFX74155 | pTM: 0.80 | Figure 2.A | HsdS1 model | 133308956 |
| HsdS3 | AFX74343 | pTM: 0.74 | Figure 2.A | HsdS1 model | 1104788464 |
| HsdM_2_HsdS1_1_ complex | M2S1_154 | ipTM: 0.65  pTM: 0.65 | Figure 2.E | NA | 1115669233 |
| HsdM_2_HsdS2_1_ complex | M2S1_155 | ipTM: 0.69  pTM: 0.69 | Figure 2.E | NA | 1072920222 |
| HsdM_2_HsdS3_1_ complex | M2S1_143 | ipTM: 0.68  pTM: 0.69 | Figure 2.E | NA | 497475310 |
| HsdM_2_Mhy1_1_ complex | M2M1 | ipTM: 0.21  pTM: 0.43 | Figure 2.E | NA | 1949096637 |
| EcoKI | M2S1_Ecoli (from EcoKI (PDB 2Y7C)) | ipTM: 0.68  pTM: 0.69 | Figure 2.E | NA | 407677113 |
| Mhy3 | AFX74157 | pTM: 0.93 | Figure 3.B | Sau3AI methyltransferase (Sau3AIM model) | 348246128 |
| Sau3AIM | Sau3AIM | pTM: 0.92 | Figure 3.B | NA | 2064436360 |
| End3 | AFX74156_End3 | pTM: 0.91 | Figure 3.B | Sau3AI restriction enzyme (PDB 4PXG) | 690703478 |
| Res3 | resIII_MH | pTM: 0.71 | Figure 4.B | Type III *M. pulmonis* restriction enzyme (Mpulmonis model) | 526706089 |
| *M. pulmonis* Mod | Mpulmonis | pTM: 0.73 | Figure 4.B | NA | 1225248911 (10.1128/jb.01669-06) |
