# Supplementary material for: Integrative multiomic characterization of the methylome and restriction-modification repertoire of Mycoplasma hyorhinis SK76
Source: Front Microbiol. 2026 Oct 22;17:1950185. doi: 10.3389/fmicb.2026.1950185 (PMC13736764; doi:10.3389/fmicb.2026.1950185)
Supplement: Supplementary file 5 [file Data_Sheet_5.DOCX]

Supplementary Material

**Supplementary Data S5. Full experimental details for LC-MS/MS DIA-NN sampling and analysis.**

Sample preparation

*M. hyorhinis* SK76 culture were grown in the conditions described before. Cell pellet was then washed twice with PBS and scraped for final collection in a 1.5 mL collection tube. Sample pellets were prepared as described in 10.1186/s12934-024-02392-3. Two biological replicates were performed. The protein concentration of each sample was measured using the Pierce BCA Protein kit following manufacturer’s protocol. Samples were prepared at a final concentration of 1 µg/ml at 10 µl and analysed at the UPF/CRG Proteomic Facility.

In-solution digestion

Samples (10 µg) were reduced with dithiothreitol (30 nmol, 37 ºC, 60 min) and alkylated in the dark with iodoacetamide (60 nmol, 25 ºC, 30 min). The resulting protein extract was first diluted to 2M urea with 200 mM ammonium bicarbonate for digestion with endoproteinase LysC (1:10 w:w, 37ºC, over 6h, Wako, cat # 129-02541), and then diluted 2-fold with 200 mM ammonium bicarbonate for trypsin digestion (1:10 w:w, 37ºC, over 8h, Promega cat # V5113).

After digestion, peptide mix was acidified with formic acid and desalted with a MicroSpin C18 column (The Nest Group, Inc) prior to LC-MS/MS analysis.

Chromatographic and mass spectrometric analysis

Samples were analyzed using an Orbitrap Astral mass spectrometer (Thermo Fisher Scientific) coupled to a Vanquish Neo liquid chromatographer (Thermo Fisher Scientific). Peptides were loaded directly onto the analytical column and were separated by reversed-phase chromatography using a 50 cm μPAC™ Neo column (Thermo Scientific, cat # COL-NANO050NEOB), featuring a structured pillar array bed with a 180 µm bed width.

Chromatographic gradient was initiated with 96% buffer A and 4% buffer B at a flow rate of 750 nL/min. Over 1.9 minutes, the percentage of buffer B was increased to 10%. The flow rate was then reduced to 250 nL/min, and the percentage of buffer B was further increased to 22.5% over 23 minutes, and then to 40% buffer B and 60% A over 11minutes. After each analysis, the column was washed for 7 min with 90% buffer B. Buffer A: 0.1% formic acid in water. Buffer A: 0.1% formic acid in water. Buffer B: 0.1% formic acid in 80% acetonitrile.

The mass spectrometer was operated in positive ionization mode with data-independent acquisition, with a full MS scans over a mass range of m/z 380-980 with detection in the Orbitrap at a resolution of 240,000. In each cycle of data-independent acquisition, 300 windows of 2 Th were used to isolate and fragment all precursor ions from 380 to 980 m/z. A normalized collision energy of 25% was used for higher-energy collisional dissociation (HCD) fragmentation. MS2 scan range was set from 150 to 2000 m/z with detection in the Astral with a maximum injection time of 3 ms.

Digested bovine serum albumin (New England Biolabs cat # P8108S) was analyzed between each sample to avoid sample carryover and to assure stability of the instrument and QCloud^1^ has been used to control instrument longitudinal performance during the project.

Data Analysis

Acquired spectra were analyzed with DIA-NN (Neural networks and interference correction enable deep proteome coverage in high throughput)^2^ (v 2.2) using a predicted spectral library from Mycoplasma hyorhinis database (as in November 2025, 84322 entries) plus a list^3^ of common contaminants. To construct a comprehensive proteome of M. hyorhinis SK76, all possible open reading frames (ORFs) were predicted using a Galaxy-based ORF finder (https://www.ncbi.nlm.nih.gov/orffinder/). Only ORFs encoding ≥20 amino acids and initiating with canonical (ATG) or alternative start codons (GTG, TTG) were retained. Stop codons were restricted to TAG and TAA, as TGA is reassigned to tryptophan in Mollicutes. Redundant sequences were filtered using CD-HIT^3^, retaining the longest representative sequence for each cluster. This approach generated a curated set of non-redundant protein sequences representing the SK76 proteome.

For peptide identification trypsin was chosen as enzyme, oxidation of methionine and N-terminal protein acetylation were used as variable modifications, whereas carbamidomethylation on cysteines was set as a fixed modification.

Precursor and fragment ion m/z mass range were adjusted to 380-980 and 150-2000, respectively. False discovery rate (FDR) was set to a maximum of 1% at both the precursor and protein level. For peptide quantification match-between-runs was enabled, protein inference was set to ‘Protein names (from FASTA)’ with ‘Heuristic protein inference’ option. The single-pass Neural Network machine learning model was used with the Quant UMS high precision quantification strategy. Default settings were used for the others parameters.

The raw proteomics data have been deposited to the PRIDE^5^ repository with the dataset identifier PXD080153.

References

1 Chiva, C. et al. QCloud: A cloud-based quality control system for mass spectrometry-based proteomics laboratories. PLoS One 13, e0189209 (2018).

2 Demichev, V., Messner, C. B., Vernardis, S. I., Lilley, K. S. & Ralser, M. DIA-NN: neural networks and interference correction enable deep proteome coverage in high throughput. Nat Methods 17, 41–44 (2020).

3 Beer LA, Liu P, Ky B, Barnhart KT, Speicher DW. (2017) Efficient quantitative comparisons of plasma proteomes using label-free analysis with MaxQuant. Methods Mol Biol. 1619:339-352.

4 Li, W., & Godzik, A. (2006). Cd-hit: a fast program for clustering and comparing large sets of protein or nucleotide sequences. *Bioinformatics*, 22(13), 1658–1659. doi:10.1093/bioinformatics/btl158

5 Perez-Riverol Y, Bai J, Bandla C, García-Seisdedos D, Hewapathirana S, Kamatchinathan S, Kundu DJ, Prakash A, Frericks-Zipper A, Eisenacher M, Walzer M, Wang S, Brazma A, Vizcaíno JA. (2022) The PRIDE database resources in 2022: a hub for mass spectrometry-based proteomics evidences. Nucleic Acids Res. Jan 7;50(D1):D543-D552.
