# Supplementary material for: Integrative multiomic characterization of the methylome and restriction-modification repertoire of Mycoplasma hyorhinis SK76
Source: Front Microbiol. 2026 Oct 22;17:1950185. doi: 10.3389/fmicb.2026.1950185 (PMC13736764; doi:10.3389/fmicb.2026.1950185)
Supplement: Supplementary file 6 [file Data_Sheet_7.ZIP › Supplementary_Data_S7/PF02976_LogoHR.pdf]

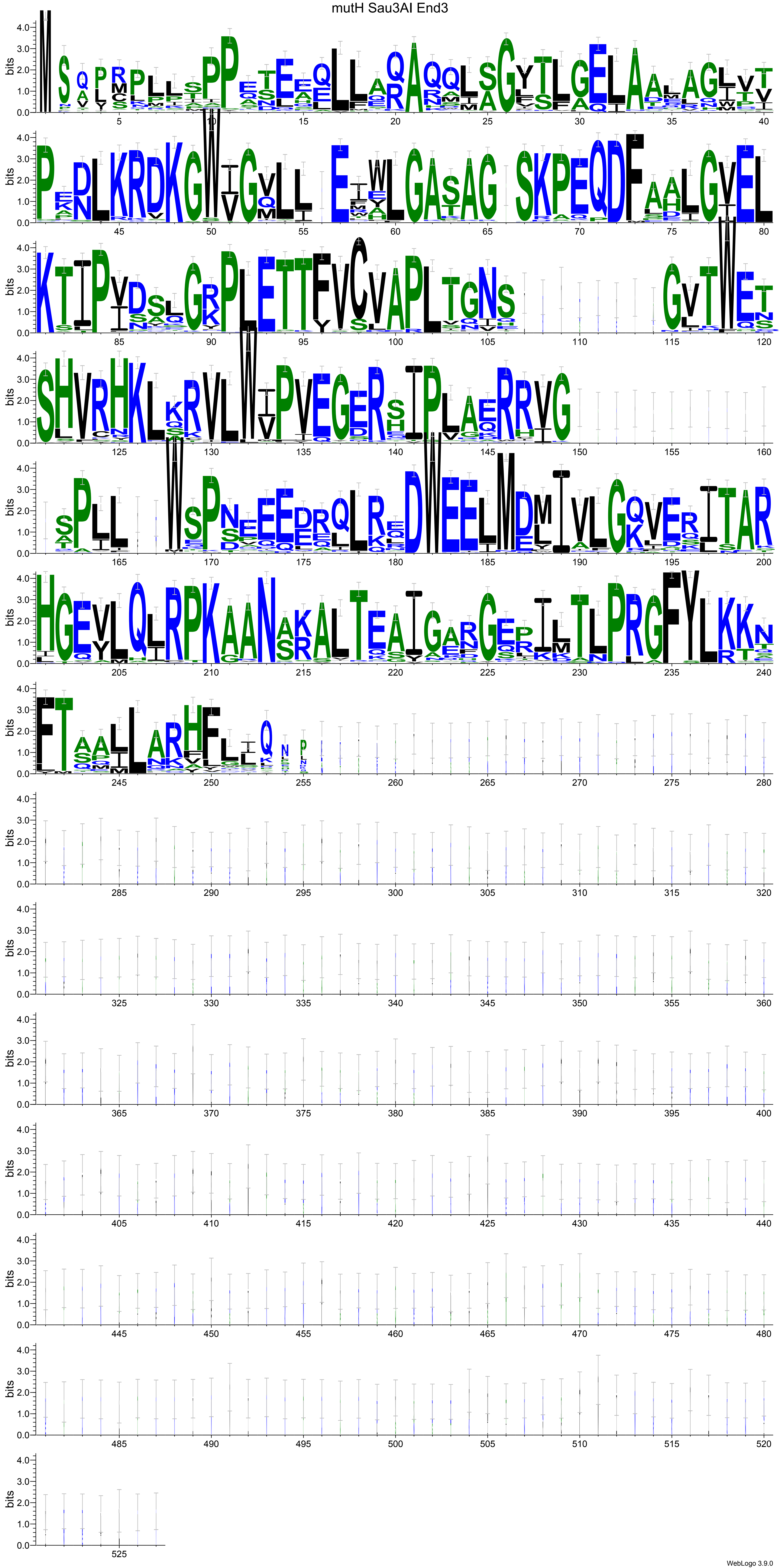
mutH Sau3AI End3
bits
WebLogo 3.9.0
