# Supplementary material for: Integrative multiomic characterization of the methylome and restriction-modification repertoire of Mycoplasma hyorhinis SK76
Source: Front Microbiol. 2026 Oct 22;17:1950185. doi: 10.3389/fmicb.2026.1950185 (PMC13736764; doi:10.3389/fmicb.2026.1950185)
Supplement: Supplementary file 7 [file Data_Sheet_8.docx]

Supplementary Material

**Supplementary Data S8. Upstream R-M system elements in *M. hyorhinis* SK76.** Promoter analysis upstream of R-M genes. Sequences show 80 bp upstream of the predicted start codon (bold). Predicted -10 Pribnow boxes in light blue, Shine-Dalgarno motifs in yellow, palindromic sequences in red. ATG start codons are underlined. i) Average expression level in arbitrary units (Table 2).
