# Supplementary material for: Integrative multiomic characterization of the methylome and restriction-modification repertoire of Mycoplasma hyorhinis SK76
Source: Front Microbiol. 2026 Oct 22;17:1950185. doi: 10.3389/fmicb.2026.1950185 (PMC13736764; doi:10.3389/fmicb.2026.1950185)
Supplement: Supplementary file 8 [file Data_Sheet_9.DOCX]

Supplementary Material

**Supplementary Data S9**. Protein IDs from the RefSeq genome (NC_019552.1) with associated RM system component, PFAM ID, and PFAM domain annotation. Only hits with e-value < 1e-5 are reported. “ND” indicates no PFAM domain detected for the protein.

| **Protein ID** | **RM component** | **PFAM ID** | **PFAM domain** |
| --- | --- | --- | --- |
| WP_015084138.1 | HsdM | PF02384 | N-6 DNA Methylase |
|  |  | PF12161 | HsdM N-terminal domain |
| WP_014335478.1 | HsdR | PF18766 | SWI2/SNF2 ATPase |
|  |  | PF22679 | Restriction endonuclease, type I, HsdR, second RecA-like helicase domain |
|  |  | PF04851 | Type III restriction enzyme, res subunit |
|  |  | PF04313 | Type I restriction enzyme R protein N terminus (HSDR_N) |
|  |  | PF00270 | DEAD/DEAH box helicase |
| WP_052306315.1 | HsdS1 | PF01420 | Type I restriction modification DNA specificity domain |
| WP_015084083.1 | HsdS2 | PF01420 | Type I restriction modification DNA specificity domain |
| WP_015084170.1 | Hsd3 | PF01420 | Type I restriction modification DNA specificity domain |
| WP_013301872.1 | Mhy1 | PF00145 | C-5 cytosine-specific DNA methylase |
| WP_013301873.1 | End1 | PF13391 | HNH endonuclease |
| WP_015084000.1 | Mhy2 | PF00145 | C-5 cytosine-specific DNA methylase |
| WP_014335361.1 | End2 | ND |  |
| WP_015084085.1 | Mhy3 | PF00145 | C-5 cytosine-specific DNA methylase |
| WP_015084084.1 | End3 | PF02976 | DNA mismatch repair enzyme MutH |
| WP_015084066.1 | Mod1 | PF01555 | DNA methylase |
|  |  | PF27727 | Uncharacterized conserved protein UCP028340 |
|  |  | PF03602 | Conserved hypothetical protein 95 (methyltransferases) |
| WP_015084065.1 | Res1 | PF04851 | Type III restriction enzyme, res subunit |
