# Supplementary material for: Integrative multiomic characterization of the methylome and restriction-modification repertoire of Mycoplasma hyorhinis SK76
Source: Front Microbiol. 2026 Oct 22;17:1950185. doi: 10.3389/fmicb.2026.1950185 (PMC13736764; doi:10.3389/fmicb.2026.1950185)
Supplement: Supplementary file 9 [file Supplementary_file_1.DOCX]

Supplementary Material

**A** Phylogenetic analysis of Type III Mod methyltransferases

**B** Reference phylogeny based on RecA protein

**Supplementary Figure S1. Phylogenetic relationships of Type III Mod methyltransferases and comparison with the bacterial species phylogeny. (A)** Maximum-likelihood phylogenetic tree of representative Type III Mod methyltransferases from diverse bacterial species, including the three larger Mod variants identified in *Mycoplasma hyorhinis* SK76. **(B)** Reference maximum-likelihood phylogeny reconstructed from the conserved housekeeping gene RecA using the same representative bacterial species. Comparison of both trees reveals that the evolutionary history of Type III Mod methyltransferases differs from the species phylogeny, consistent with the dynamic evolution of restriction–modification systems through gene gain, loss and horizontal gene transfer. Bootstrap support values are indicated at the nodes, and branch lengths represent the number of substitutions per site.
