# Supplementary material for: Integrative multiomic characterization of the methylome and restriction-modification repertoire of Mycoplasma hyorhinis SK76
Source: Front Microbiol. 2026 Oct 22;17:1950185. doi: 10.3389/fmicb.2026.1950185 (PMC13736764; doi:10.3389/fmicb.2026.1950185)
Supplement: Supplementary file 10 [file Supplementary_file_2.DOCX]

Supplementary Material

**Supplementary Figure S2. Conservation of Type III R–M system genes across *M*. *hyorhinis* strains.** Sequence conservation of *mod* and *res* genes across multiple *M. hyorhinis* strains. Color intensity reflects both sequence identity and alignment coverage: dark blue indicates >90% identity with >75% coverage, light blue indicates 70–90% identity with >75% coverage, and red indicates <70% identity or <75% coverage. This representation highlights both the presence of homologous genes and their degree of sequence conservation.
