# Supplementary material for: Integrative multiomic characterization of the methylome and restriction-modification repertoire of Mycoplasma hyorhinis SK76
Source: Front Microbiol. 2026 Oct 22;17:1950185. doi: 10.3389/fmicb.2026.1950185 (PMC13736764; doi:10.3389/fmicb.2026.1950185)
Supplement: Supplementary file 11 [file Supplementary_file_3.DOCX]

Supplementary Material

**Supplementary Figure S3. Detection of N6-methyladenine (6mA) in genomic DNA by dot blot analysis.** Representative dot blot showing genomic DNA (gDNA) from *Escherichia coli* DH5α, the commercial *dam⁻/dcm⁻* strain (NEB) used as an unmethylated control, and *M. hyorhinis* SK76 (MH). DNA samples were spotted onto a nylon membrane in serial three-fold serial dilutions and probed with an anti-6mA antibody. Dashed circles indicate the positions where DNA was applied to the membrane. The experiment was performed using three independent biological replicates, each analyzed with two technical replicates. Bottom, quantitative densitometric analysis of the dot blot experiments performed using Fiji. The numbers correspond to the integrated density measured for each spot, and the heatmap color scale reflects the relative signal intensity, with darker red indicating lower values and darker blue indicating higher values.
